# Identification and Analysis of Anticancer Therapeutic Targets from the Polysaccharide Krestin (PSK) and Polysaccharopeptide (PSP) Using Inverse Docking

**DOI:** 10.3390/molecules29225390

**Published:** 2024-11-15

**Authors:** Carlos Iván López-Gil, Alejandro Téllez-Jurado, Marco Antonio Velasco-Velázquez, Miguel Angel Anducho-Reyes

**Affiliations:** 1 Department of Biotechnology, Universidad Politécnica de Pachuca, Zempoala 43830, Mexico; krlosviralcero@gmail.com (C.I.L.-G.); alito@upp.edu.mx (A.T.-J.); 2 Department of Pharmacology, Faculty of Medicine, Universidad Nacional Autónoma de México, Coyoacan 04510, Mexico; marcovelasco@unam.mx

**Keywords:** PSK, PSP, inverse docking, carcinogenic proteins, *Trametes versicolor*, bioinformatics, immunostimulant

## Abstract

The natural compounds PSK and PSP have antitumor and immunostimulant properties. These pharmacological benefits have been documented in vitro and in vivo, although there is no information in silico which describes the action mechanisms at the molecular level. In this study, the inverse docking method was used to identify the interactions of PSK and PSP with two local databases: BPAT with 66 antitumor proteins, and BPSIC with 138 surfaces and intracellular proteins. This led to the identification interactions and similarities of PSK and the AB680 inhibitor in the active site of CD73. It was also found that PSK binds to CD59, interacting with the amino acids APS22 and PHE23, which coincide with the rlLYd4 internalization inhibitor. With the isoform of the K-RAS protein, PSK bonded to the TYR32 amino acid at switch 1, while with BAK it bonded to the region of the α1 helix, while PSP bonded to the activation site and the C-terminal and N-terminal ends of that helix. In Bcl-2, PSK interacted at the binding site of the Venetoclax inhibitor, showing similarities with the amino acids ASP111, VAL133, LEU137, MET115, PHE112, and TYR108, while PSP had similarities with THR132, VAL133, LEU137, GLN118, MET115, APS111, PHE112, and PHE104.

## 1. Introduction

The basidiomycete fungus *T. versicolor* is a cosmopolitan organism of forest ecosystems worldwide. Its presence is reported with greater frequency in temperate, woodland, boreal, and tropical forests [1], where it grows on fragile or dead trees and dry trunks [2]. In China and Japan, it is prized for its nutritional and medicinal value, and the diverse bioactive compounds it contains [3]. Specifically, its medicinal value through the ingestion of infusions, extracts, or powders has led to the recognition of a broad range of physiological activities, including immunomodulator, anticancer, antioxidant, and hepatoprotective effects [4,5,6,7,8]. Studies have demonstrated that these physiological activities are related mainly to bioactive components like polysaccharopeptide (PSP) and polysaccharopeptide-K (PSK) [9,10,11,12,13,14,15]. These two polysaccharopeptides are similar due to the presence of glycosidic bonds β-1,3, β-1,4 and β-1,6 [16,17] but differ in their chemical structure since PSP contains rhamnose and arabinose, while PSK contains fucose, fructose, galactose, mannose, and xylose. Partial or total acid hydrolysis can generate a variety of polysaccharides with lower molecular weights, such as disaccharides and D-glucose monosaccharides [18,19], that may show structural and topological similarity for they share molecular fingerprints with other D-glucans from distinct sources [20,21,22]. In this context, structural analyses of compounds like CVG (*Coriolus versicolor* glucan) [6], CVP (*Coriolus versicolor* polysaccharides) [23], laminarin [24], and sizofiran [25] could reveal shared structural, molecular, and topological characteristics with PSK, PSP, and their hydrolases that could be utilized in more detailed docking analyses and digital molecular fingerprinting studies that may relate them to antitumor and immunomodulator activities.

In cancer treatments, extracts of PSK and PSP obtained from the cultivated mycelia of *T. versicolor* have been utilized as complementary adjuvants, alone or combined with radio- or chemotherapy. The pharmacological and therapeutic benefits of this approach have been documented in vitro and in vivo by means of cell cultures, experimental animal models, and clinical assays that confirm their antitumor, immunostimulant, and antioxidant properties [26]. As a result of clinical assays, the immunomodulator action of PSK has been proposed as an agent that can induce the expression of the interleukin-8 gene (IL-8) in peripheral blood mononuclear cells (PBMCs) after oral administration by stimulating T cell proliferation and enhancing the function of T CD4+ cells in the intestine and associated lymphoid tissue [27,28,29,30], while also significantly prolonging the survival of patients with stomach, colorectal, and lung cancer [31,32,33].

Clinical assays with PSP extracts suggest that this substance acts as an immunomodulator by fostering the proliferation of splenocytes and T and B cells in rodents, and monocytes (CD14^+^/CD16) in humans. Some studies have documented, biochemically, that administration reduces the expression of the Fas lymphocyte receptor and increases monocyte counts [34,35,36,37]. Other research has revealed that administering PSP extracts can inhibit the proliferation of diverse cancer cells through the activation of immune cells by increasing the expression of cytokines and chemokines, like tumor necrosis factor-α (TNF-α), interleukins IL-1β and IL-6, histamine, and prostaglandin E, increasing the infiltration of T and dendritic cells into tumors, and improving the quality of life of patients who suffer adverse effects of chemotherapy [38]. In vitro studies, in turn, have demonstrated that applying PSP extracts in MDA-MB-231 breast cancer and LNCaP prostate cancer cell lines significantly reduced proliferation [39]. This result has also been observed in PC-3 and DU-145 prostate cancer cell lines, though with a less efficacious level of suppression [40]. In addition, the direct toxicity of PSP has been documented in the MCF-7, HBL-100, T-47D, and Walker 256 cell lines of breast cancers, the A-549 and SWi573 lines of lung cancers, and the LoVo and LS174-T lines of colon cancers [41].

Today, in silico experimental modeling can be used to refine, increase, and bring together data generated by in vivo and in vitro experimental models to enhance our understanding of the pharmacology of synthetic or natural molecules and associate them with cellular, genetic, and molecular processes. Inverse molecular docking is an in silico experimental modeling technique that can be used to predict the possible interactions of a specific group of proteins with a ligand or pharmacophore when the function of the ligand is known, but that of the receptors is not. These features allow researchers to simulate, infer, and elucidate possible action mechanisms. It can also help identify possible side effects of new medications, or aid in choosing the least harmful treatment regimen for a certain disease [42].

For some 50 years, researchers have focused their studies on the biological, therapeutic, and pharmacological effects of D-glucans [43] in in vivo and in vitro model systems, but the structural characteristics and molecular interactions of D-glucans via in silico methodologies have seldom been examined. Therefore, the present study was designed to utilize PSK and PSP, polypeptide saccharides extracted from the basidiomycete fungus *Trametes versicolor*, and β-D-glucans with structural, molecular, and topological similarity, as ligands in the search for, and analysis of, potential therapeutic targets in cancerous activity, using the computerized tool of inverse docking and by designing two databases.

## 2. Results

### 2.1. Inverse Molecular Docking

**Figure 1:**
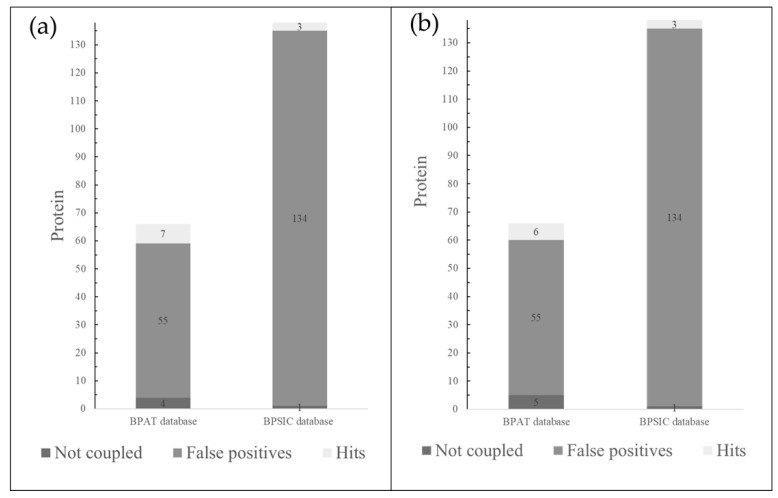
(**a**). Bar graphs show the hits found in the database of antitumor proteins (BPAT) and the database of intracellular and surface proteins (BPSIC) with the PSK ligand. (**b**) Bar graphs show the hits found for BPAT and BPSIC with the PSP ligand.

The two databases, BPAT with 66 proteins and BPSIC with 138 proteins, were subjected to, and analyzed by, the set of Ligand-Based Target Searching (LBTS) scripts. This produced 10 and 9 target proteins, respectively, for the PSK (Figure 1a) and PSP ligands (Figure 1b). The target proteins showed energy docking values less than or equal to −8.5 Kcal/mol, the value that was used as a limit or general cut-off limit to reduce the occurrence of false positives [44]. Of the total of 19 hits obtained, 11 were discarded: 6PEB, 5EU3, 5EU4, and 5EU5 because they shared interactions with PSP and PSK, 5EU6 and IPH52 because they interacted only with PSK, and 5EA0, which interacted only with PSP. The 6PEB hit that corresponded to a metabolic enzyme (NAMPT) was omitted due to insufficient information on its crystallographic quality for later docking analysis, while the 5EU3, 5EU4, 5EU5, and 5EU6 hits were discarded because they represented a group of crystals of the same protein and corresponded to class I HLA histocompatibility antigens of synthetically designed peptidic ligands altered with the goal of increasing immunogenicity [45].

**Figure 2:**
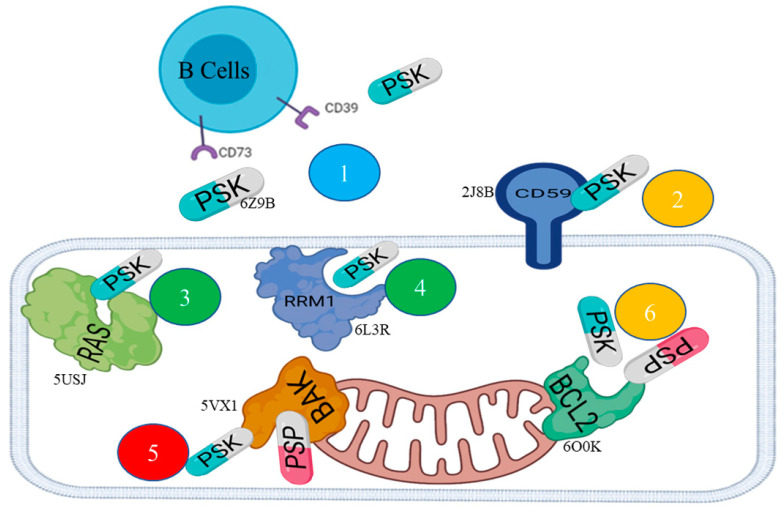
General diagram of the interaction of the PSP and PSK ligands with diverse extracellular and intracellular proteins. The action mechanisms with the hits or target proteins are shown in different colors: blue = immunostimulatory; orange = apoptosis resistance; green = proliferation; red = apoptosis.

Table 1 shows the results of the analysis of the in silico modeling by means of inverse docking. Each hit presents the PDB (Protein Data Bank) codes with their respective docking energy values for PSP and PSK, the type of protein, and its possible function or mechanism in cancerogenic processes. The graph in Figure 2 shows the action mechanisms of the PSP and PSK ligands on the hits or therapeutic targets. The pose clustering analysis of the models chosen from eight complexes (Table 1) showed as a result that the total threshold average was −6.3 Kcal/mol. The highest ratio of acceptable poses, in relation to calculating RMSD, was 17/20 for the 6L3R-PSK and 5VX1-PSK complexes, while for the 2J8B-PSK, 5USJ-PSK, and 6O0K-PSK complexes the ratio of acceptable poses was 13/20. The rest of the complexes ranged from 9/20 to 12/20 (Supplementary Material Table S1).

After selecting and analyzing each hit, it was important to add estimates from the calculations of docking energy, glycosidic torsion angles, and intrinsic energy of carbohydrates. The Vina-carb program was used for this operation. The results of the in silico modeling of specific docking delimited the grid box of the interaction sites identified previously, showing optimized docking energy values between −7.6 and −6.3 (Table 1). The lengths of the hydrogen bonds were measured using the measuring tool of the PyMOL program, considering the criterion of a length limit of 3.5Å. No result of the length measurements of the hydrogen bonds for the eight hits with PSK and PSP exceeded this limit, as they presented values between 1.2 and 3.2 Å. Finally, 2D diagrams were constructed using the Discovery Studio 2021 program. They show the results of the visualization and identification of the shared Van der Waals and π-π interactions with the ligands.

### 2.2. Hit 1, Bcl-2 Apoptosis Regulatory Protein (6O0K)

#### 2.2.1. PSK-Bcl-2

**Figure 3:**
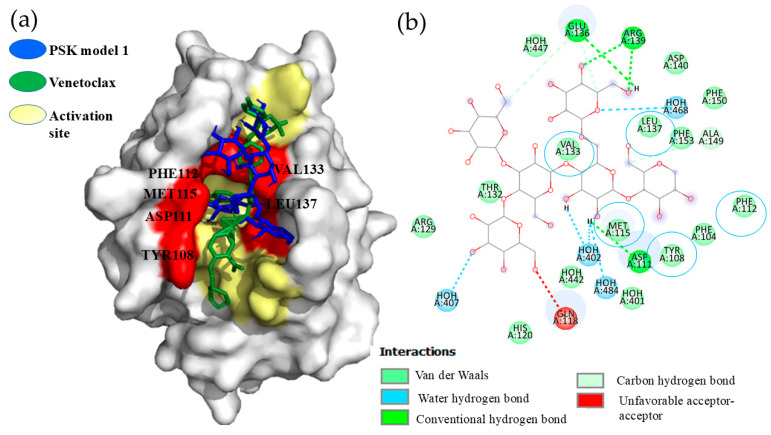
(**a**) Model 1, PSK bonded at the Bcl-2 activation site and Venetoclax interactions bonded at the Bcl-2 activation site. (**b**) Model 1, interactions of PSK at the Bcl-2activation site.

All the PSK-Bcl-2 interaction models were below the cut-off limit of −6.0 Kcal/mol, with minimal spatial variations. The comparative analysis of PSK interaction with Bcl-2 amino acids, and with respect to the pharmaceutical inhibitor, Venetoclax, demonstrated that PSK bonded inside the delimited interaction area of Venetoclax and the BH3 peptide, shown in Figure 3a. In addition, we observed interactions by Van der Waals forces in the VAL133, LEU137, MET115, ASP111, PHE112, TYR108, and PHE104 amino acids, where the similarities of PSK and Venetoclax were found in the VAL133, LEU137, MET115, PHE112, and TYR108 amino acids, with a hydrogen bond in the APS111 amino acid, as Figure 3b shows. These results show inhibition of the Bcl-2 protein.

#### 2.2.2. PSP-Bcl-2

The results of the docking analyses of all the PSP-Bcl-2 models were below the cut-off limit of −6.0 Kcal/mol, in a range of −6.2 to −6.8 Kcal/mol. Although adequate affinity values were achieved, observations showed that models 1–5, 8, and 9 did not bind at Venetoclax’s binding site. In contrast, models 6 and 7 did succeed in bonding there. Model 6 presented interactions by Van der Waals forces in the THR132, VAL133, LEU137, GLN118, MET115, APS111, PHE112, and PHE104 amino acids, while model 7 showed interaction by a hydrogen bond in the GLN118 amino acid, and by Van der Waals forces in the THR132, VAL133, LEY137, ARG146, MET115, ASP111, PHE112, TYR108, and PHE104 amino acids.

### 2.3. Hit 2, CD59 Human Antigen (2J8B)

**Figure 4:**
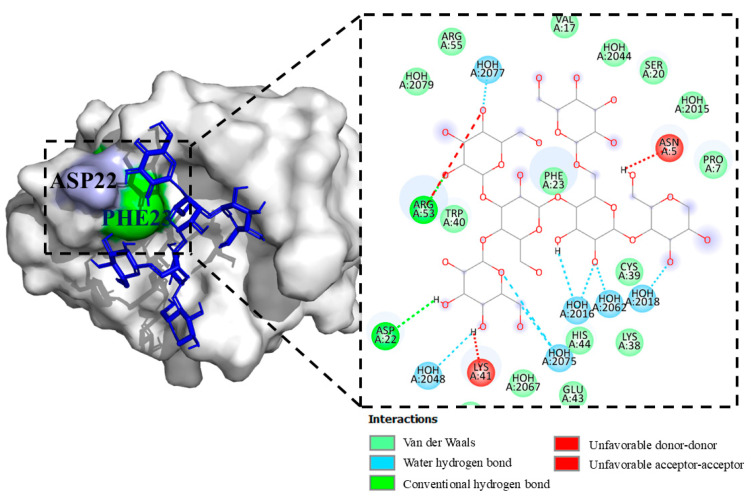
Model 1, CD59-PSK complex and its hydrogen bonding interactions (blue) and Van der Waals interactions (green).

Figure 4 shows the results of the interaction of PSK with the protein. Of the nine interaction models, only model 1 exceeded the interaction limit value of −6.0 Kcal/mol. In model 1 of the CD59-PSK complex, hydrogen bond interactions were found in the amino acids ASP22 and ARG53, and by Van der Waals forces in the amino acids PRO7, VAL17, SER20, PHE23, LYS38, CYS39, TRP40, GLU43, and HIS44. Two negative interactions per donor–donor were also found in the LYS41 amino acid and one per acceptor–acceptor in the ASN5 amino acid.

### 2.4. Hit 3, K-RAS Isoform (5USJ)

**Figure 5:**
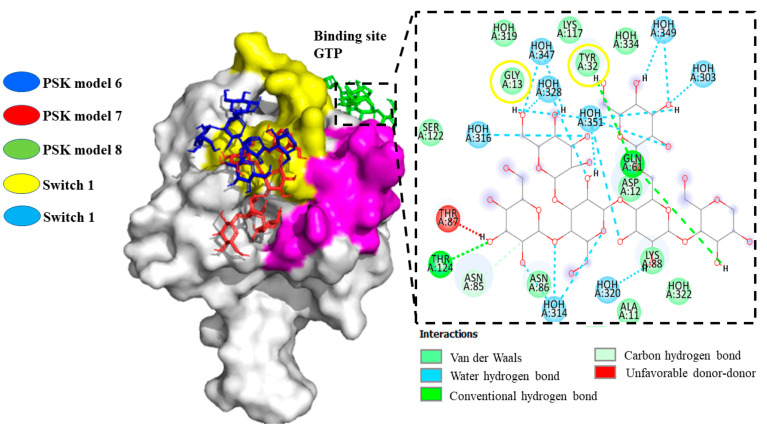
Models 6–8 of PSK interactions with the crystalline structure of the K-RAS isoform (5USJ); model 8, PSK interactions at the GTP binding site. The surface of switch 1 is highlighted in yellow and the surface of switch 2 is highlighted in purple.

The results of the docking analysis of PSK-K-RAS interaction demonstrated that the PSK ligand in models 1–5 bonded to an allosteric site with a binding energy of −5.6 to −6.4 Kcal/mol. Observations showed that it did not bond to switch 1, which forms the binding interface of the RAS effector and regulatory proteins (GAP and GEFs), which bond specifically to amino acids reported as participants in inhibiting their functionality (PRO34, ASP33, TYR32) [46]. The same result was obtained for switch 2, an allosteric sac that controls GTP (Guanosine Triphosphate) affinity and effector interactions, without bonding to amino acids that participate in inhibiting its functionality (GLN61, ALA59) for the intermediate space of the two interrupters (ASP38) and the P-loop (GLY12, GLY13) [47]. However, models 3–5 and 9 showed PSK bonding at the active site of the isoform, though their energy was not more negative or equal to the cut-off limit of −6.0 Kcal/mol. For model 8, we observed the generation of a hydrogen bond with amino acid TYR32 and a Van der Waals electrostatic interaction with amino acid GLY13, specifically at the binding site of GTP or activation of RAS. Models 6 and 7 interacted with the surface of switch 1 (Figure 5).

**Figure 6:**
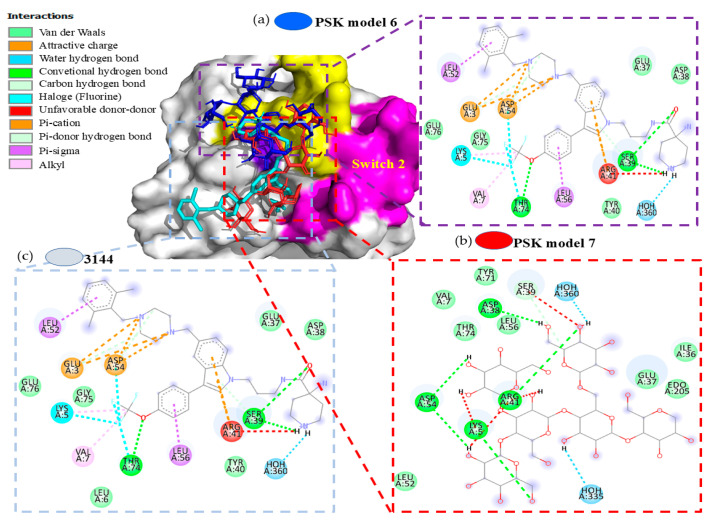
Models 6 and 7, superposition of the 3144 and PSK molecules at switch 1 of K-RAS. (**a**) Interactions of the K-RAS-3144 compound. (**b**) Model 6, interactions of the K-RAS-PSK compound. (**c**) Model 7, interactions of the K-RAS-PSK compound.

To complement our analysis of models 6 and 7, we performed comparative docking utilizing the commercial molecule 3144 (Pan-RAS-IN-1), which was designed to be directed to multiple adjacent sites in switches 1 and 2 through compounds like dichlorophenol, indole, trifluoromethoxy, and piperidine−4-carboxamide, so it is considered a multivalent inhibitor [48] (Figure 6a) that interacts with switch 1. The interaction results of model 6 showed similarities through hydrogen bonds in the amino acids SER39, TYR40, and ARG41, and Van der Waals interactions in the amino acids GLU37 and ASP38 (Figure 6b). Model 7 presented similarities through a hydrogen bond in the amino acids ASP38, SER39, ARG41, and ASP54, and Van der Waals interactions in the amino acids GLU37 and THR74 (Figure 6c) [49]. It also showed shared interactions between PSK and 3144 with the amino acid SER39 of switch 1 of the K-RAS protein. The interaction energies in both models were not more negative than the cut-off limit of −6.0 Kcal/mol.

### 2.5. Hit 4, Ribonucleotide Reductase Enzymes (6L3R)

Although we obtained models that were more negative than the cut-off limit of −6.0 Kcal/mol, only models 7 and 8 showed bonding to PSK. It was not possible, however, to detect interactions at the catalytic site, where PSK binds to the guanosine diphosphate substrate (GDP), since the key interaction amino acids with this substrate are THR624, SER625, THR209, GLU441, CYS225, ASN437, CYS439, and CYS 462 [50].

### 2.6. Hit 5, Bak Protein (5VX1)

#### 2.6.1. PSK-Bak

**Figure 7:**
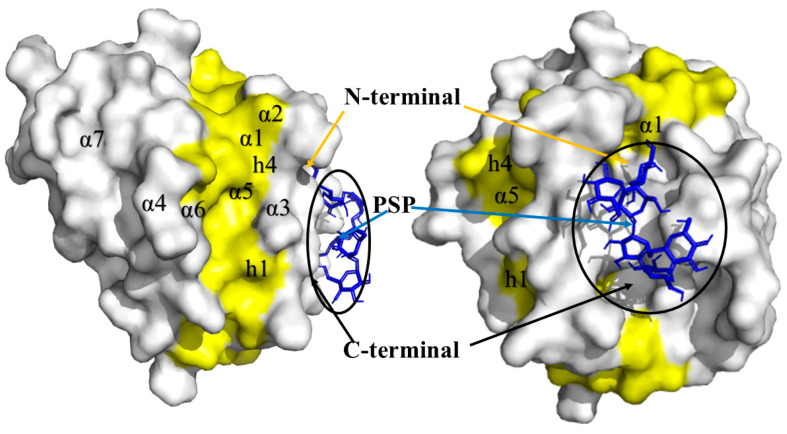
PSK-Bak binding complex in α1 helix.

The analysis of PSK-Bak interactions was carried out with the nine models generated by docking. These models presented minimal variation, with energy values more negative than the cut-off limit of −6.0 Kcal/mol. All nine models allowed us to observe a shared hydrogen bond with the amino acid ARG42 and a Van der Waals interaction in the region of helix α1 (Figure 7).

#### 2.6.2. PSP-Bak

**Figure 8:**
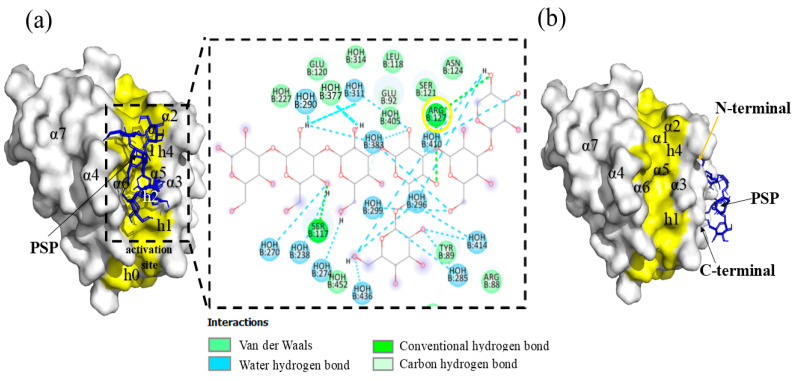
(**a**) Model 9, PSP-Bak interaction complex at the activator site. (**b**). Model 1, PSP-Bak binding complex in α1 helix. The surface of the activator site is highlighted in yellow and the PSP molecule is shown in blue.

In the analysis of the interactions of the Bak protein with the PSP molecule, all models presented energies that were more negative than the cut-off limit of −6.0 Kcal/mol, in a range of −6.6 to −7.8 Kcal/mol. Models 1 to 8 presented minimal spatial variations, but model 9 showed the formation of a hydrogen bond in amino acid ARG127, and the generation of Van der Waals interactions in amino acids ILE85, ARG88, GLU92, TYR89, LEU118, GLU120, SER121, and ASN124 (Figure 8a) in the zone of the activation site, specifically inside the h0-h4 hydrophobic residue groove.

### 2.7. Hit 6, the CD73 Protein (6Z9B)

**Figure 9:**
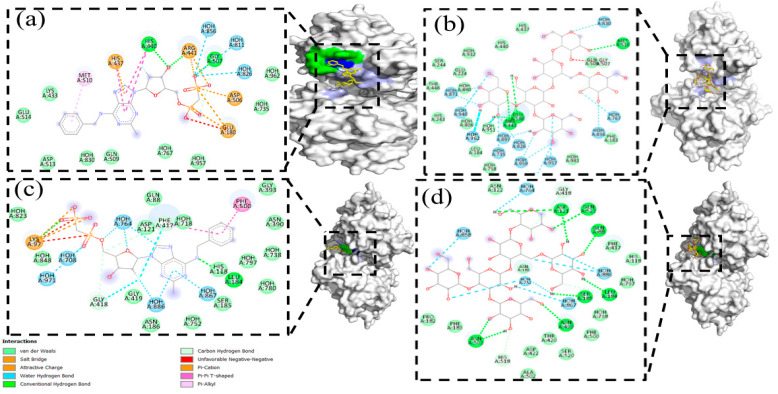
(**a**) Image of the 6Z9B-AB680 complex; the inhibitor binds to the active site, hydrogen bonding interactions are shown in green, Van der Waals interactions in yellow, and π-π interactions in red. (**b**). Image of the 6Z9B-PSK complex; PSK binds to the active site, interactions with amino acids are shown in green. (**c**). Image of the 6Z9B-AB680 complex; the inhibitor binds to an allosteric site, hydrogen bonding interactions are shown in green and π-π interactions in red (**d**). Image of the 6Z9B-PSK complex; PSK binds to an allosteric site, hydrogen bonding interactions are shown in green.

To carry out the docking analysis of PSK and obtain a parameter for comparison, we utilized the AB680 compound as a reference. This compound is catalogued as a selective CD73 inhibitor. It is currently being evaluated in phase 1 clinical assays [51]. The results of the docking analysis of AB680 with the CD73 protein showed an affinity energy of −7.3 Kcal/mol with interactions by a hydrogen bond in amino acids HIS437, HIS440, ARG441, GLY507, ASP506, and GLU180, Van der Waals interactions in amino acids GLU514, LYS433, ASP513, and GLN509, and π-π interactions in amino acid MET510 (Figure 9a). Figure 9b shows the result of model 1 for the docking analysis of PSK with the best affinity energy value (−6.3 Kcal/mol). The comparative analysis showed shared interactions of the hydrogen bonds for both molecules in amino acids at the active sites ARG441, GLU180, and GLY507, with a difference of three hydrogen bonds for the AB680 molecule in the amino acids HIS440, HIS437, and ASP506. Although PSK did not manifest π-π interaction in the amino acid MET510, an interaction of the hydrogen bond was observed.

Another interesting result of the docking analysis of AB680 and model 1 of PSK was the interaction of both at an allosteric site of CD73 (Figure 9c,d) with interaction energies of −8.4 and −6.8 Kcal/mol, respectively. The interactions of the hydrogen bonds of both molecules were common in amino acids LEU184 and GLY418. Other important interactions occurred in amino acid PHE417 with a Van der Waals interaction for PSK and a hydrogen bond for AB680, and in amino acid PHE500 with a Van der Waals interaction for PSK and a π-π bond for AB680.

**Figure 10:**
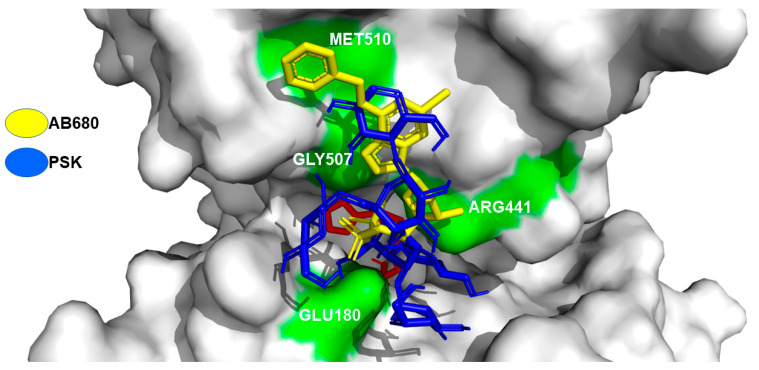
Model 1, superimposition of the AB680 inhibitor and PSK in the active site of the transformation of adenosine phosphate to adenosine of the CD73 protein. Amino acid interaction surface (green).

Figure 10 depicts the superposition of the AB680 compound and the PSK molecule at the active transformation site of adenosine phosphate to adenosine in the CD73 protein. Similar interactions were observed in amino acids GLU180, ARG441, GLY507, and MET510.

Our second database included 700 molecules that presented a molecular identity or Tanimoto index ≥ 0.90 in the β-(1–3), β-(1–4), and β-(1–6) bonds, indicative of structural similarity to the base of D-glucose. This database was used to perform a virtual screening analysis with hit CD73, considered due to the nature of its active site that presents affinity to saccharides. The results showed that only 9 of those 700 molecules bonded specifically to the active site (Supplementary Material Table S2).

## 3. Discussion

The results of this study allowed us, using reverse molecular docking, to identify and analyze potential mechanisms of interaction proteins and the polysaccharide peptides PSP, PSK, and other β-D-glucans, that could be used in the treatment of cancer.

The comparative interaction analysis denoted that PSP binds to most of the amino acids with which the Venetoclax inhibitor interacts, suggesting PSP’s potential to act as an inhibitor of the Bcl-2 protein, thereby promoting apoptosis and the death of cancer cells. Bcl-2 belongs to the family of pro- and anti-apoptotic proteins that control cell survival by interrupting apoptosis and facilitating tumor development. Hence, it is a key therapeutic target for developing activators that favor apoptosis and cell death [52]. Some clinical assays using Venetoclax, a highly selective oral Bcl-2 inhibitor, have demonstrated apoptosis- or cell death-promoting activity in Bcl-2-dependent, malignant hematological neoplasms, especially cases of chronic lymphocytic leukemia (LLC) [53,54]. In conclusion, the comparative analysis of the interactions of models 6 and 7 demonstrated that PSP shares an interaction of Van der Waals forces with the Venetoclax inhibitor in the PHE112 amino acid. This comparative interaction analysis indicates that PSP binds to most of the amino acids with which the Venetoclax inhibitor interacts, suggesting PSP’s potential to act as an inhibitor of the Bcl-2 protein, thereby promoting apoptosis and the death of cancer cells.

With respect to the CD59 human antigen protectin, or the membrane inhibitor of reactive lysis (MIRL), which is a cell surface protein anchored to the cell membrane by glycosylphosphatidylinositol (GPI), it functions to protect lysis host cells by binding to C5b8 and C5b9 compounds to inhibit the formation of C9 polymeric during the final steps of the membrane attack compound (MAC) [55,56]. The fourth domain—consisting of 114 amino acids—of the intermedilysin ILY (rlLYd4), a specific cytotoxin of human cells like the cytolytic factor obtained from *Streptococcus intermedius*, permits the inhibition of CD59 by forming a compound that penetrates into the cell where it is degraded through the action of lysosomes [57]. In this context, PSK could function in a manner similar to rlLYd4 and perform a function of intracellular entrance into CD59. Wickham et al. [58] reported diverse amino acid interactions between ILY and CD59 that respect PSK-CD59 model 1. Similar interactions were observed as a hydrogen bond in the APS22 amino acid and as Van der Waals forces with the PHE23 amino acid. This leads to the inference that the similarities between the rlLYd4 molecule and PSK in the different amino acids of the CD59 protein could make it possible to internalize the protein inside the cell or initiate receptor-mediated endocytosis.

The in silico results of the comparative interaction of a natural molecule against a chemically designed one like 3144, and the one that projects as an inhibitor of the RAS protein upon binding to the surface of switch 1, altered the active state of the mutant protein to an off state, causing the inhibition of tumor growth and of the viability of cells of pancreatic, colon, and lung cancer [59], suggesting PSK’s anticancer effect by presenting an interaction with the surface of switch 1 in K-RAS. The mutant RAS oncogene is associated with approximately 30% of all human cancers. Some reports indicate that it is expressed in three isoforms—K-RAS, H-RAS, and N-RAS—with high sequence homology. K-RAS is the isoform expressed most frequently [60,61]. It is classified as a hydrolase, specifically of the GTPasa group. This protein hydrolyzes its natural substrate of GTP to GDP, causing the transmission of diverse signals from the exterior of the cell into its interior. These signals are transduced as cell growth, differentiation, migration, and proliferation factors that function as interrupters activated by GTP, bind to the active site, and are turned off by GDP. Recent descriptions show that the mutant K-RAS protein presents two possible modifications in the GLY12 amino acid by CYS12 or ASP12 that alter their behavior which, compared to the function of the native protein, is maintained in a permanently active state that causes neoplasias related primarily to colorectal and lung cancer [62]. The foregoing indicates the importance of these results, for they mean that it is feasible to perform in vitro experiments directed specifically to this type of target protein.

The ribonucleotide reductase proteins (RNRs), or ribonucleoside diphosphate reductases (rNDPs), are enzymes that belong to the family of the oxidoreductases. Their principal function is to catalyze the reductions in purine and pyrimidine ribonucleotides to their corresponding deoxyribonucleotides [63], which are the basic units for the replication and reparation of the DNA of eucaryotic cells. However, studies have documented that greater RNR activity is associated with malignant and metastatic transformations of cancer, because one of its fundamental characteristics is uncontrolled cell proliferation [64]. It has been reported, as well, that cancer cells are subject to a metabolic reprogramming of glucose that reduces ATP production but foments that of macromolecules for cell replication, including dNTP. In this way, the inhibition of these enzymes leads to a reduction in intracellular dNTP concentrations, an inhibition of the synthesis and reparation of DNA, the detention of the cell cycle, and the promotion of apoptosis. In this regard, the discovery, design, and development of RNR inhibitors could constitute viable treatment options as monotherapies or combined with cancer chemotherapy. Recent advances in cancer biology will allow the greater development of RNR inhibitors with enhanced efficacy and reduced toxicity for treating numerous forms of cancer [65]. The regulation of the RNR protein occurs through the transfer of a free tyrosyl radical at the catalytic site (site C). It is regulated transcriptionally by allosteric sites [66], though the results of docking indicate that PSK does not have interaction at the allosteric sites, despite the fact that interactions are observed at site C.

The apoptosis mechanism is highly orchestrated to allow the destruction of damaged and abnormal cells that may be generated during both normal and pathological physiological conditions. The alteration of apoptosis allows the development of tumors and makes tumor cells more resistant to conventional cytotoxic therapies. The decisive phase of apoptosis is regulated mainly by the Bcl2 protein family, which is made up of anti-apoptotic (Bcl-2, Bcl-XL, Bcl-W, MCL-1, BFL-1, BCL-B and A1) and pro-apoptotic (Bak, Bax, Bad, Bad, Bid, Bik, Blk, BimL, PUMA, NOX, BMF, HRK) molecules. Specifically, Bak and Bax play key roles in producing mitochondrial disfunction and apoptotic cell death. Bak is an integral protein membrane present in the cytosolic faces of mitochondria and the endoplasmic reticulum, while Bax must be translocated from the cytosol after an apoptotic stimulus. The formation of Bak homo- or heterodimers is an important mechanism in inducing apoptosis [67,68]. For this reason, researchers have focused efforts on the search for, and the design and development of, diverse molecules that can activate this protein. Descriptions indicate that its activation site is formed by a hydrophobic groove made up of helixes α3, α4, and α5 and hydrophobic residues (h0–h4) [69]. This result demonstrates an action similar to the bonding of specific monoclonal anti-BAK1 antibodies that bond to the C-terminal and displace helix α1 [70].

The interaction of PSP in this area could be indicative of an activating action of the Bak protein, since the peptide-RT (Bim-RT) acts by bonding to Bak’s canonic hydrophobic groove (α3, α4, α5), principally through hydrophobic residues (h0–h4) (Figure 8b) and a conserved saline bridge (ARG127), where PSP binds by a hydrogen bond to these same amino acids and, through contact, binds to helixes α3, α4, and α5. This initiates the permeabilization of the external mitochondrial membrane (MMP), though this has yet to be demonstrated in vitro, utilizing cytochrome C as the biomarker during the activation of Bak [71]. This may demonstrate a possible inhibitory action, since upon comparing the amino acid interaction we noted a charge–charge interaction coincidence in amino acids R42 (ARG42) in helix α1 [72]. Inside the structure of helix α1, a C-terminal interaction and an N-terminal interaction were observed. The C-terminal interaction indicates a secondary activation due to the displacement of helix α1 of the Bak protein.

Various studies have demonstrated that enzymes CD73 and CD39 are responsible for generating an immunosuppressor environment characterized by high production of the nucleoside adenosine that promotes evasion by the tumor or favors the development and progression of cancer cells. Our in silico analysis confirmed that the PSK ligand presents a potential strategy for interrupting this pathway of tumor resistance by blocking the active site. This would prevent the hydrolysis of immunogenic ATP in the immunosuppressor adenosine [52,73,74].

The results of the static and flexible docking analysis of the nine molecules of β-D-glucans and protein CD73 demonstrated interaction energies and optimized ligands in the flexible analysis. The comparative analysis of the flexible docking of the nine β-D-glucans with the natural adenosine monophosphate substrate and the commercial inhibitor AB680 generated similar values for interaction energies and ligand efficiency (LE) of −6.3 to −7.5 and −0.095 to −0.3, respectively. In conclusion, the molecule with the best characteristics of interaction energies and ligands in this analysis was CVG, so it is the leading candidate as an inhibitor of the CD73 protein.

In relation to this, a bibliographic review carried out by Novack and Vetvicka [75] revealed the diversity of data that exist on comparisons of the structure, molecular size, and biological effect of the β-D-glucans from distinct biological sources. For example, they described the compound schizophillan, which presents antitumor activity, supposedly due to the presence of a triple helix topology and a molecular weight above 100 kDa [76]. However, some descriptions suggest that the alkaline treatment used in extraction and purification procedures destroys this structure, leading to the conclusion that the triple-helix structure is likely not the only efficacious form of β-glucan [77,78,79]. Moreover, a high molecular weight and ramification of β-D-glucans are not necessary for them to be biologically active. Those authors also described the discovery by Kabat et al. [80], who established that the size of the polysaccharide determinants of antigens at the binding site of an antibody corresponds to six or seven monosaccharide units. The K-Ras hit was omitted from this analysis because its interaction occurs at an allosteric site and it requires molecules that are structurally larger and more diverse.

In all the molecular docking analyses, it was very important to use physiological pH conditions between 7.35 and 7.45 in order to simulate a human blood environment. Niu et al. [81], in their work, performed modeling of molecular docking interactions using Acetylcholinesterase (AChE) and compounds with high and low cholinesterase inhibitory capacity. The objective was to analyze the effect of the ionization state of these inhibitors on the amino acid residues of the AChE active site under the conditions of human blood at physiological pHs of 7.0 and 7.4. The in silico molecular docking results showed that some of these inhibitory compounds did not exhibit hydrogen bond interactions with the AChE active site at a physiological pH of 7.4, which differed from the results obtained from these same molecules at a physiological pH of 7.0. This allowed them to consider that the chemical structures of these inhibitors should be optimized to increase their activity at a physiological pH of 7.4.

## 4. Materials and Methods

### 4.1. Computer Methodology

#### Specifications of the Computer Equipment

We used a desktop PC with the following characteristics: Intel CoreI9 processor (Intel^®^, Santa Clara, CA, USA), 10,900, 5.2 GHz with 10 physical cores, two 8 GB, 3200 MHz RAM XPG modules (ADATA Technology, New Taipei City, Taiwan), a 500 B solid state unit, and a graphics card with at least 2 GB of VRAM. The scripts required were designed, modified, and executed with the aid of various dependencies in Python3 language (https://www.python.org/downloads/ accessed on 15 January 2023). This allowed the installation of pdb tools (http://www.bonvinlab.org/pdb-tools/ accessed on 15 January 2023) and the MGLTools library (https://ccsb.scripps.edu/mgltools/downloads/ accessed on 15 January 2023) for the manipulation and analysis of files in PDB format. Once the necessary files were generated, docking was performed using Autodock Vina (https://vina.scripps.edu/downloads/ accessed on 20 January 2023). Finally, Vina-carb software (https://glycam.org/docs/othertoolsservice/downloads/downloads-software/index.html accessed on 20 January 2023) was used to improve the results obtained from the interactions between the target proteins and the PSK and PSP polysaccharides and β-D-glucans with structural, molecular, and topological similarity. It was employed as a platform to conduct graphic modeling and perform the visualization and analysis of the molecular interactions between the proteins and the PSK and PSP polysaccharides and other β-D-glucans.

### 4.2. Preparation of the Ligands

The molecules of PSK, PSP, and β-D-glucans, aside from the reference ligands Venetoclax, 3144 (Pan-RAS-IN-1), and AB680, were downloaded from the PubChem database (https://pubchem.ncbi.nlm.nih.gov/ accessed on 22 January 2023) in SDF format to extract their SMILES (Simplified Molecular Input Line Entry System) codes (Supplementary Material Information S1).

The data of the SMILES codes were loaded into the platform (http://www.cheminfo.org/Chemistry/Cheminformatics/FormatConverter/index.html accessed on 22 January 2023), adding parameters of coordinates in 3D in Merck’s MMFF94 force field [82], at a physiological pH of 7.4. The files containing the SMILES codes and parameters were run with Autodock tools v1.5.6 to assign a rotation root using the Torsion tree tool. The final files were saved in pdbqt format to perform the docking processes with the Autodock Vina 1.1.2 program.

### 4.3. Protein Database

Two local databases were created, the first with antitumor proteins (BPAT), the second with surface and intracellular proteins (BPSIC). The files with the structural information of these proteins were obtained using the Advanced Search Query Builder of the RCSB PBD platform (https://www.rcsb.org/ accessed on 22 January 2023) [83], with the keywords cancer and Homo sapiens, as well as the attributes of the experimental method of X-ray diffraction and a refinement resolution lower or equal to 2.5Å (Supplementary Material Database S1).

### 4.4. Reverse Molecular Docking

Identification and analysis of interactions were performed on the 9 default models generated between each one of the interactions of the target proteins and the PSK and PSP ligands and β-D-glucans, utilizing a blind, inverse molecular docking procedure with the set of Ligand-Based Target Search scripts (LBTS) (https://github.com/zhenglz/LBTS accessed on 25 January 2023) [84], which were incorporated into an inverse docking. The pose clustering analysis was performed in Autodock Vina using a cut-off value ≤ 3.0 Å. A single cluster was generated with the docked models, which were compared with the lowest energy structure considering the calculated RMSD values of the ligand, to observe the stability and repeatability of its positioning.

### 4.5. Molecular Docking with Vina-Carb

The results of the blind, inverse molecular docking modeling that presented cut-off values more negative than, or equal to, the limit of −8.5 Kcal/mol, were then subjected to a docking analysis utilizing the Vina-carb complement script. This generated 9 interaction models, which were used for identification and analysis, considering a new cut-off value, more negative than, or equal to, −6.0 Kcal/mol, as is recommended for sites delimited by a specific grid box. The Vina-carb script adds characteristics of carbohydrate intrinsic energy (CHI) [85] with specific energy profiles (chi_coeff = 1). This makes it possible to penalize the magnitude of the CHI and produce more precise models. In addition, using the CHI cut-off parameter (chi_cutoff = 2) maintains the minimum optimal energy and quantifies the preferences of the glycosidic torsion angles so as to not generate penalization, thus improving the bond of the compound and its interaction energy [86].

### 4.6. Docking Analysis

The protein–ligand compounds were examined with the Analyze tools of the Autodock4 tools program (https://autodock.scripps.edu/download-autodock4/ accessed on 27 January 2023). This permitted identifying and quantifying the interactions of the ligand’s hydrogen bonds with the amino acids of the protein. The lengths of these interactions were measured with the PyMOL v 2.5 program (https://pymol.org/2/ accessed on 27 January 2023). The 2D compounds were visualized with the Show 2D diagram tool of the Discovery Studio 2021 program—academic version (https://discover.3ds.com/discovery-studio-visualizer-download accessed on 30 January 2023) to identify, visualize, and analyze the conventional and unfavorable interactions of the hydrogen bonds, Van der Waals, and π-π interactions. Interactions in the protein–ligand compounds were identified using a nomenclature that indicated the location of amino acids according to the protein sequence and a 3-character abbreviation for the amino acids that interacted with the ligand IUPAC (ej:ARG210).

### 4.7. Virtual Screening

The second database was constructed with the structural information of the PSK, PSP, CVG, CVP, and other D-glucan compounds reported and contained in the PubChem platform (https://pubchem.ncbi.nlm.nih.gov/ accessed on 3 February 2023). SMILES codes obtained from the Open Babel platform (https://www.cheminfo.org/Chemistry/Cheminformatics/FormatConverter/index.html accessed on 3 February 2023) [87] were used to conduct the search for these protein–ligand compounds. Later, considering the criterion of structural similarity or molecular fingerprinting [88] in the β-(1–3), β-(1–4), and β-(1–6) bonds, as well as similar biological activity, a Tanimoto coefficient > 0.90 was used [89].

## 5. Conclusions

The analysis of these results and other molecular docking experiments using cholinesterase inhibitors with different structures suggested that their activity is affected by the use of different physiological pH values, which could help optimize drug design. In addition, it has been described that an important factor to consider during molecular docking or quantitative structure–activity relationship (QSAR) modeling is the water molecules found in the crystal structures, which contribute to the shape and flexibility of the site binding, mainly when hydrogen bonds are generated between proteins and their ligands. For example, some works report improvement in the accuracy of the ligands, a reduction in false positives, and an improvement in docking performance by up to 20% [90]. It is necessary to mention that there is a lack of adequate literature to establish a cut-off value for polysaccharide–protein interactions. However, the most negative cut-off value or equal to the limit of −8.5 Kcal/mol, used in this study as a filter on the interactions of PSP and PSK polysaccharides and target proteins, was selected based on the results presented by Zhang et al. [44]. In this study, they proposed a novel computational pipeline for a high-throughput ligand target search against the user-defined structure database to predict the potential targets of three herbal ingredients, acteoside, quercetin, and Epigallocatechin-3-gallate (EGCG), in human structural proteome. In this sense, although acteoside and quercetin are chemically different from PSP and PSK, they belong to the group of glycosides, that is, they generally contain monosaccharides, a criterion that we used to establish the cut-off value of −8.5 Kcal/mol in the process and analysis of inverse molecular docking modeling.

In this context, the inverse docking methodology, complemented with Vina-carb, made it possible to identify six individual or shared hits of the PSK and PSP compounds, as well as nine molecules with the structural nature of β-D-glucans that interacted with human proteins involved in tumor and cancer processes. Based on these results, it is feasible to consider that the number of experimental targets that can be tested in vitro and in vivo is reducible. Specifically, it was possible to identify inhibition interactions in the targets mAb CD73 and CD59, and in the mutant K-RAS oncogene, by interrupting processes related to carcinogenesis and tumor formation. Moreover, we succeeded in isolating key interactions in the activation of Bak and inhibition of Bcl-2 proteins that trigger the mechanisms of apoptosis and cell death in cancer cells. All these interactions obtained by means of in silico analysis demonstrate the mechanisms of the PSK and PSP molecules—two important components of the cell wall of *T. versicolor*—as anticancer and antitumor agents, thus confirming their therapeutic and pharmaceutical value.

## Figures and Tables

**Table 1:** Selected hits of virtual projection using inverse docking.

Hits	Codes	Scoring (Kcal/mol)		Protein Type	Mechanism
		PSK	PSP		
1	6O0K	−6.5	−6.8	Bcl-2 apoptosis regulator	Promotes anti-apoptosis
2	2J8B	−6.3		Membrane-bound glycoprotein	Protects host cells from lysis
3	5USJ	−6.3		Mutant KRAS G12D	Active molecular switch regulators that increase the capacity for invasion and metastasis, and decrease apoptosis
4	6L3R	−7.2		RRM1: large subunit of ribonucleoside–diphosphate reductase	RRM1 participates in regulating cell proliferation
5	5VX1	−7.6	−7.8	BAK	Initiates oligomerization and permeabilization of the outer mitochondrial membrane
6	6Z9B	−6.3		Hydrolases	Hydrolyzes ATP (Adenosine Triphosphate) and AMP (Adenosine Monophosphate) to generate adenosines, which inhibit the immune response

## Data Availability

The original contributions presented in this study are included in the article and Supplementary Materials; further inquiries can be directed to the corresponding author/s.

## Supplementary Materials

The following supporting information can be downloaded at: https://www.mdpi.com/article/10.3390/molecules29225390/s1, Table S1. Clustering analysis, Information S1. Preparation of the ligands, Database S1. Protein database, Table S2. Static and flexible docking analysis of chemical structures with molecular similarity to PSK and PSP.
