# Supplementary material for: Identification and Analysis of Anticancer Therapeutic Targets from the Polysaccharide Krestin (PSK) and Polysaccharopeptide (PSP) Using Inverse Docking
Source: Molecules. 2024 Nov 15;29(22):5390. doi: 10.3390/molecules29225390 (PMC11596896; doi:10.3390/molecules29225390)
Supplement: Supplementary file 1 [file molecules-29-05390-s001.zip › molecules-3249205-supplementary.pdf]

Table S1. Clustering Analysis

Complex	6O0K-PSK		6O0K-PSP		2J8B-PSK		5USJ-PSK	
Model	Affinity (Kcal/mol)	RMSD	Affinity (Kcal/mol)	RMSD	Affinity (Kcal/mol)	RMSD	Affinity (Kcal/mol)	RMSD
1	-7	0	-7.4	0	-6.4	0	-6.3	0
2	-6.9	2.27	-7.2	1.537	-6	2.21	-6.1	5.351
3	-6.5	1.95	-7.1	3.369	-5.8	2.044	-6	2.429
4	-6.4	2.406	-7.1	3.613	-5.6	2.452	-6	3.101
5	-6.4	1.694	-7.1	2.793	-5.5	2.43	-6	1.69
6	-6.4	1.399	-7	1.761	-5.5	2.007	-6	20.526
7	-6.4	10.42	-7	3.28	-5.5	3.744	-5.9	2.969
8	-6.3	10.088	-7	2.778	-5.5	2.684	-5.9	2.809
9	-6.2	8.051	-7	3.013	-5.4	3.172	-5.9	2.644
10	-6.2	2.504	-6.9	5.132	-5.4	4.26	-5.8	2.526
11	-5.9	2.326	-6.8	2.776	-5.1	1.922	-5.8	2.532
12	-5.9	10.418	-6.8	1.46	-5	2.073	-5.7	2.587
13	-5.9	2.631	-6.8	4.003	-4.9	2.969	-5.7	2.673
14	-5.8	2.731	-6.7	3.167	-4.8	3.345	-5.6	5.774
15	-5.8	9.662	-6.7	4.488	-4.8	3.157	-5.6	2.77
16	-5.7	2.626	-6.7	4.34	-4.7	3.242	-5.5	19.987
17	-5.6	11.92	-6.5	4.375	-4.7	1.707	-5.5	8.822
18	-5.6	2.449	-6.4	5.796	-4.6	2.151	-5.4	19.413
19	-5.4	2.973	-6.4	4.337	-4.5	1.652	-5.3	2.712
20	-5.4	9.851	-6.4	5.326	-4.5	4.37	-5.3	16.145
**Acceptable poses**	**13**		**9**		**13**		**13**	
**Average threshold (Kcal/mol)**	**-6.2**		**-7**		**-5.3**		**-5.8**	

Complex	6L3R-PSK		5VX1-PSK		5VX1-PSP		6Z9B-PSK		
Model	Affinity (Kcal/mol)	RMSD	Affinity (Kcal/mol)	RMSD	Affinity (Kcal/mol)	RMSD	Affinity (Kcal/mol)	RMSD	
1	-7.1	0	-7.6	0	-8.5	0	-6.7	0	
2	-7	0.943	-7.4	1.831	-8.4	2.156	-6.3	1.815	
3	-6.8	1.298	-7.3	2.083	-8.3	2.29	-6.2	3.113	
4	-6.6	2.686	-7.1	1.823	-8	2.018	-6.1	2.067	
5	-6.6	2.077	-6.8	2.947	-8	3.058	-6.1	1.565	
6	-6.4	1.729	-6.3	3.046	-7.9	3.595	-6.1	3.494	
7	-6.3	3.429	-6.2	2.793	-7.9	2.002	-6	3.461	
8	-6.3	2.888	-6.2	2.551	-7.8	2.628	-6	3.972	
9	-6.3	1.757	-6.2	2.09	-7.8	2.748	-5.9	2.552	
10	-6.2	1.978	-6.1	2.799	-7.7	3.363	-5.9	2.346	
11	-6.2	1.774	-6.1	2.815	-7.6	3.6	-5.9	2.748	
12	-6.1	2.063	-6.1	3.124	-7.6	3.305	-5.8	3.181	
13	-6.1	2.258	-6.1	2.589	-7.5	1.474	-5.7	3.49	
14	-6.1	3.258	-6	2.764	-7.5	1.679	-5.6	3.313	
15	-6.1	2.465	-6	2.544	-7.4	2.989	-5.5	2.797	
16	-6	2.53	-5.8	2.702	-7.4	2.577	-5.4	2.436	
17	-6	2.836	-5.7	1.426	-7.4	3.299	-5.3	2.762	
18	-5.9	2.1	-5.6	3.257	-7.4	3.75	-5.3	4.09	
19	-5.9	2.182	-5.6	2.905	-7.3	3.438	-5.2	2.314	
20	-5.8	3.187	-5.5	2.855	7.3	3.431	-5.1	4.212	
**Acceptable poses**	**17**		**17**		**12**		**11**		**Average total threshold (Kcal/mol)**
**Average threshold (Kcal/mol)**	**-6.3**		**-6.3**		**-7.9**		**-5.8**		**-6.3**

Note: The cells colored in gray represent the values of RMSD ≤ 3.0.

Information S1 Preparation of the ligands

PSK

PubChem SID: 135316798; **Registration of substances:** 134192-05-5; **MW:** 931.52 g/mol, 100kDa; **Composition:** C36H19O30; **SMILES:**[C]1([C](Or[C]([C]([C]1O[C]1[C]([C]([C]([C](O1)[C]Or)Or)Or)Or)Or)Or[C]1[C]([C]([C](Or[C]1[C]Or[C]1[C]([C]([C]([C](O1)[C]Or)Or)Or)Or)Or[C]1[C]([C]([C]Or[C]1[C]Or)Or)Or)Or)Or)[C]Or)Or[C]1[C]([C]([C]([C](O1)[C]Or)Or)Or)Or

PSP

PubChem SID: Not available; **Registration of substances:** Not available; **MW:** 1102.60 g/mol, 100kDa; **Composition:** C42H22O36; **SMILES:**[C]1([C]([C](Or[C]([C]1O)[C]Or[C]1[C]([C]([C]([C](O1)[C]Or)Or)Or)Or)Or[C]1[C]([C](Or[C]([C]1O)[C]Or)Or[C]1[C]([C](Or[C]([C]1O)[C]Or)Or[C]1[C]([C](Or[C]([C]1O)[C]Or)[Or])Or)Or)Or)Or)Or[C]1[C]([C]([C]([C](O1)[C]Or)Or)Or[C]1[C]([C]([C]([C](O1)[C]Or)Or)Or)Or)Or

CVG

PubChem CID: 2108; **Registration of substances:** Not available; **MW**: 194.18 g/mol; **Composition:** C7H4O6; **SMILES:** [C]1([C](Or[C]([C]([C]1O)Or)[C]Or)Or[C])Or

CVP

PubChem SID: Not available; **Registration of substances:** Not available; **MW**: 483.27; g/mol; **Composition:** C18H11O16; **SMILES:**[C]1(Or[C]([C]([C]([C]1O)Or[C]1[C]([C]([C]([C](O1)[C]Or)Or)Or)Or)Or)[C]Or)Or[C]1[C]([C](Or[C](Or)[C]1O)[C]Or)Or

Reference ligands

Venetoclax

PubChem CID: 49846579; **Registration of substances:** Not available; **MW:** 868.4; **Composition**: $C_{45}H_{50}CIN_7O_7S$; **SMILES:**CC1(CCC(=C(C1)C2=CC=C(C=C2)Cl)CN3CCN(CC3)C4=CC(=C(C=C4)C(=Or)NS(=Or)(=Or)C5=CC(=C(C=C5)NCC6CCOCC6)[N+](=Or)[Or-])OC7=CN=C8C(=C7)C=CN8)C

3144 (Pan-RAS-IN-1)

PubChem CID: 102004330; **Registration of substances:** Not available; **MW:** 717.66; **Composition**: $C_{36}H_{41}CI_2F_3N_6O_2$; **SMILES:**C1CNCCC1(C(=Or)NCCCN2C=C(C3=C2C=CC(=C3)CN4CCN(CC4)CC5=C(C=CC=C5Cl)Cl)C6=CC=C(C=C6)OC(F)(F)F)N

AB680

PubChem CID: 155809269; **Registration of substances:** Not available; **MW**: 578.81; **Composition**: $C_{20}H_{22}CIFN_4O_9P_2^{-2}$; **SMILES:**CC(C1=CC=CC=C1F)NC2=CC(=NC3=C2C=NN3C4C(C(C(O4)COP(=Or)(CP(=Or)(Or)[Or-])[Or-])Or)Or)Cl

Database S1. Protein database

1A7S, 1AGW, 1AQV, 1AQW, 1AQX, 1B1E, 1B1I, 1B1J, 1B8Y, 1BD8, 1BHS, 1BJ1, 1BLX, 1BMK, 1BOZ, 1C83, 1C84, 1CAQ, 1CIZ, 1CLL, 1CLU, 1CS3, 1D3G, 1D3H, 1D4A, 1D5R, 1DEB, 1DGB, 1DGF, 1DGG, 1DGH, 1E3G, 1EAX, 1EC6, 1ECV, 1EXA, 1EXX, 1F5K, 1F5L, 1FCX, 1FCY, 1FCZ, 1FDS, 1FDT, 1FH0, 1FVC, 1FVT, 1FY3, 1GMY, 1GS4, 1GUX, 1H00, 1H01, 1H07, 1H08, 1H1P, 1H1R, 1H1S, 1H59, 1H66, 1H69, 1H7S, 1HCK, 1HCL, 1HVY, 1HW3, 1HW4, 1I5R, 1I72, 1I79, 1I7B, 1I7C, 1I7M, 1J55, 1JAH, 1JAI, 1JCQ, 1JCZ, 1JD0, 1JDH, 1JQH, 1JTV, 1K3A, 1K8U, 1K96, 1K9K, 1K9P, 1KBC, 1KHX, 1KI0, 1KLG, 1KLU, 1LGP, 1LGQ, 1LQB, 1MAZ, 1MEJ, 1MEN, 1MEO, 1MMP, 1MMQ, 1MMR, 1MP8, 1MQ4, 1MQB, 1MZC, 1N0W, 1N26, 1N4M, 1N4Q, 1NWU, 1OC0, 1OES, 1OET, 1OEV, 1OKY, 1P5Z, 1P60, 1P61, 1PYE, 1PYO, 1PYX, 1Q0B, 1Q2U, 1Q3D, 1Q3W, 1Q41, 1Q91, 1Q92, 1QIA, 1QIC, 1QR1, 1QX3, 1R0P, 1R1W, 1R2, 1R2D, 1R2E, 1R2I, 1RHJ, 1RV1, 1RVD, 1S1P, 1S1R, 1S2A, 1S2C, 1S63, 1S9W, 1S9Y, 1SA4, 1SC8, 1SPJ, 1SU3, 1T15, 1T29, 1T64, 1T67, 1TN3, 1TSR, 1TTM, 1TUP, 1TVB, 1TVH, 1UNG, 1UNH, 1UNL, 1UOL, 1UPK, 1UU3, 1UU7, 1V1K, 1VJ9, 1VJA, 1VKG, 1VPP, 1VYW, 1VYZ, 1W0X, 1W0Z, 1W10, 1W11, 1W12, 1W13, 1W14, 1W98, 1WCH, 1WER, 1WMA, 1WQ6, 1WYX, 1XF0, 1XI2, 1XJB, 1XKK, 1XOW, 1XPZ, 1XQ0, 1XQ3, 1XQC, 1XRJ, 1XUC, 1XWS, 1YC1, 1YC3, 1YC4, 1YCS, 1YER, 1YES, 1YET, 1YPV, 1YUC, 1YW7, 1YW9, 1YY8, 1YZ1, 1Z5M, 1Z8G, 1Z95, 1ZIV, 1ZQ5, 1ZS0, 1ZSP, 1ZTE, 1ZUA, 1ZUQ, 1ZVX, 2AC0, 2ADQ, 2ADU, 2AHI, 2ANG, 2AO6, 2AOA, 2AOB, 2ARY, 2ASU, 2ATA, 2AX6, 2AX7, 2AX8, 2AX9, 2AXA, 2AXN, 2BBA, 2BDF, 2BDG, 2BIK, 2BIM, 2BIN, 2BIO, 2BIP, 2BIQ, 2BKF, 2BNQ, 2BNR, 2BNU, 2BO9, 2BZH, 2BZI, 2BZJ, 2BZK, 2BZS, 2C3I, 2C6C, 2C6D, 2C6E, 2C6G, 2C6P, 2CBZ, 2CIJ, 2CLX, 2CN5, 2D06, 2D1X, 2D8N, 2DUV, 2E0T, 2ETL, 2F4J, 2F53, 2FBT, 2FBV, 2FBX, 2FBY, 2FC0, 2FGB, 2FMM, 2FVD, 2FWY, 2FWZ, 2G1T, 2G2H, 2G9N, 2GCN, 2GCO, 2GCP, 2GEE, 2GQG, 2GSS, 2GT9, 2GTW, 2GTZ, 2GUO, 2GV6, 2GV7, 2GVG, 2GVJ, 2H55, 2H8H, 2H9G, 2HD5, 2HLE, 2HQU, 2HXK, 2HYI, 2HZ5, 2HZQ, 2HZR, 2I3H, 2I3I, 2I9A, 2IAL, 2IEJ, 2IF5, 2IFQ, 2ILR, 2IMS, 2IMT, 2IOG, 2IOK, 2ITN, 2ITV, 2IUW, 2IW6, 2IW8, 2IW9, 2J0S, 2J1W, 2J1X, 2J1Y, 2J1Z, 2J8B 2J20, 2J21, 2J2I, 2J4Z, 2JAV, 2JCN 2JDO, 2JDR, 2JDS, 2JDT, 2JDV, 2JIH, 2NL9, 2NMN, 2NMO, 2NN8, 2NS2, 2NTE, 2NWG, 2NWN, 2NYR, 2O88, 2OCJ, 2OO0, 2OOT, 2OPM, 2OVH, 2OZ7, 2P1T, 2P1U, 2P1V, 2P4K, 2P5E, 2P5W, 2P85, 2PCU, 2PE4, 2PIO, 2PIP, 2PIQ, 2PIR, 2PIT, 2PIU, 2PIV, 2PIX, 2PJL, 2PKL, 2PL3, 2PN7, 2PQK, 2PSQ, 2PVF, 2PVY, 2PWL, 2PX6, 2PY3, 2PYE, 2PZ1, 2PZ5, 2PZP, 2Q3E, 2Q7I, 2Q7J, 2Q7K, 2Q7L, 2Q81, 2QBX, 2QCC, 2QCD, 2QCE, 2QCF, 2QCG, 2QCH, 2QCL, 2QCM, 2QCN, 2QFO, 2QG0, 2QG2, 2QG4, 2QMS, 2QXH, 2QXI, 2QXJ, 2R2W, 2R3F, 2R3G, 2R3H, 2R3I, 2R3J, 2R3K, 2R3L, 2R3M, 2R3N, 2R3O, 2R3P, 2R3Q, 2R3R, 2R64, 2RA4, 2RBH, 2RF5, 2RFS, 2RH1, 2RIQ, 2RKU, 2TCL, 2UUH, 2UUI, 2UVL, 2UVM, 2UWD, 2UZI, 2UZR, 2UZS, 2V1Y, 2V4B, 2V5Q, 2V5W, 2V5X, 2V8W, 2V8X, 2V8Y, 2V9R, 2V9S, 2V9T, 2VCI, 2VKI, 2VM5, 2VM6, 2VPF, 2VSL, 2VUK, 2VVK, 2VWF, 2VX3, 2W0G, 2W0J, 2W0Z, 2W17, 2W18, 2W1H, 2W3L, 2W5A, 2W5B, 2W5H, 2W7X, 2W83, 2W97, 2WA0, 2WD2, 2WD3, 2WEV, 2WGJ, 2WGP, 2WGX, 2WI1, 2WI2, 2WI3, 2WI4, 2WI5, 2WI6, 2WKM, 2WMQ, 2WMR, 2WMV, 2WMW, 2WMX, 2WND, 2WOG.2X2R, 2WOR, 2WOS, 2WTV, 2X0U, 2X0V, 2X0W, 2X29, 2X39, 2X4Z, 2X7C, 2X7D, 2X7E, 2X7S, 2X7T, 2X7U, 2XAB, 2XBJ, 2XCV, 2XCW, 2XCX, 2XDK, 2XDL, 2XDS, 2XDU, 2XDX, 2XEF, 2XEG, 2XEI, 2XEJ, 2XHR, 2XHT, 2XJB, 2XJC, 2XJD, 2XJE, 2XJF, 2XJG, 2XJJ, 2XJX, 2XK2, 2XMR, 2XMS, 2XOC, 2XOZ, 2XP0, 2XP2, 2XP3, 2XP4, 2XP5, 2XP6, 2XP7, 2XP8, 2XP9, 2XPA, 2XPB, 2XT3, 2XUS, 2XWC, 2XWR, 2Y6E, 2Y6W, 2Y96, 2YAD, 2YBG, 2YGN, 2YGO, 2YGP, 2YHD, 2YI0, 2YI6, 2YI7, 2YIQ, 2YIR, 2YIT, 2YJ1, 2YLP, 2YLQ, 2YXJ, 2Z7Q, 2Z7R, 2Z7S, 2ZGV, 3A99, 3AOX, 3APA, 3ARN, 3AT2, 3AT4, 3AU4, 3AYA, 3AYC, 3AYD, 3AYE, 3B1U, 3B24, 3B25, 3B26, 3B27, 3B28, 3B7G, 3B97, 3B9S, 3BBB, 3BBC, 3BBF, 3BCH, 3BER, 3BET, 3BGM, 3BH8, 3BH9, 3BHB, 3BHT, 3BHU, 3BHV, 3BIN, 3BIY, 3BJF, 3BO8, 3BOR, 3BXM, 3BZ3, 3C0Y, 3C0Z, 3C10, 3C39, 3C3A, 3C3B, 3C3C, 3C4E, 3C4F, 3C4H, 3C5F, 3C5G, 3C5R, 3C7P, 3C7Q, 3CBM, 3CBO, 3CBP, 3CBX, 3CBY, 3CBZ, 3CC0, 3CE4, 3CEK, 3CJF, 3CJG, 3CQV, 3CRY, 3CS4, 3CS6, 3CS9, 3CSH, 3CSI, 3CSJ, 3CWE, 3CZD, 3D1M, 3D7D, 3D7F, 3D7G, 3D7H, 3D7V, 3D8W, 3D9S, 3D9Z, 3DAI, 3DAZ, 3DBU, 3DC3, 3DC9, 3DCC, 3DCS, 3DCW, 3DD0, 3DEY, 3DJH, 3DJI, 3DKP, 3DU8, 3E1R, 3E5A, 3E7A, 3E7B, 3E7G, 3E87, 3EAH, 3EAX, 3EB1, 3EKO, 3EKR, 3ENE, 3ESK, 3EU7, 3EX7, 3F7G, 3F7H, 3F7I, 3F81, 3FC2, 3FDL, 3FDM, 3FE7, 3FEA, 3FEC, 3FED, 3FEE, 3FF3, 3FFD, 3FHB, 3FQN, 3FQR, 3FQT, 3FQU, 3FQW, 3FQX, 3FUG, 3G03, 3G1R, 3G4I, 3GCP, 3GCQ, 3GCS, 3GCU, 3GCV, 3GD8, 3GFT, 3GGF, 3GJF, 3GNI, 3GRW, 3GSS, 3GUS, 3H0V, 3H0W, 3H3B, 3H7W, 3H82, 3HB4, 3HB5, 3HHU, 3HI9, 3HKN, 3HKQ, 3HKT, 3HKU, 3HL5, 3HLJ, 3HMO, 3HMP, 3HPJ, 3HXT, 3HY3, 3HY6, 3IE3, 3IK8, 3IKD, 3IKG, 3INM, 3INQ, 3IO8, 3IO9, 3ITK, 3IX0, 3JPV, 3JSX, 3JUT, 3K1X, 3K2L, 3KAB, 3KAC, 3KAD, 3KAF, 3KAG, 3KAH, 3KAI, 3KCE, 3KCZ, 3KFX, 3KJ0, 3KJ1, 3KJ2, 3KJD, 3KJF, 3KJN, 3KJQ, 3KLA, 3KLM, 3KPZ, 3KQ6, 3KR3, 3KRR, 3KUQ, 3KZ0, 3KZD, 3KZE, 3L11, 3L54, 3L95, 3L9L, 3L9M, 3L9N, 3LBZ, 3LDL, 3LDN, 3LDO, 3LDP, 3LDQ, 3LE4, 3LE6, 3LLP, 3LQ8, 3LQ9, 3LXK, 3LXL, 3LXP, 3M3Z, 3MA3, 3MAX, 3MB6, 3MB7, 3ME3, 3MHJ, 3MHK, 3MK8, 3MTR, 3MTU, 3MXV, 3MXW, 3MYJ, 3N1F, 3N1G, 3N1M, 3N1V, 3N45, 3N46, 3N5E, 3N5G, 3N5H, 3N5J, 3N5N, 3N6K, 3NBH, 3NBI, 3NBQ, 3NCL, 3NMQ, 3NMW, 3NMX, 3NS9, 3NSZ, 3O0I, 3O33, 3O35, 3O36, 3O37, 3O3A, 3O3B, 3O3D, 3O3E, 3O3U, 3O50, 3O64, 3O7W, 3ODK, 3OGU, 3OOB, 3OSE, 3OVM, 3OWH, 3OWJ, 3OWK, 3OWL, 3OWX, 3OX1, 3OX2, 3OX3, 3OY8, 3OZJ, 3P0N, 3P0P, 3P0Q, 3P0Y, 3P2T, 3P53, 3P5A, 3P8F, 3P8G, 3P8X, 3P92, 3P95, 3PB1, 3PCV, 3PE1, 3PE2, 3PE4, 3PH9, 3PJ8, 3PO6, 3POZ, 3PP0, 3PQZ, 3PY7, 3Q01, 3Q05, 3Q2R, 3Q2U, 3Q6S, 3Q6U, 3Q6W, 3QDJ, 3QF9, 3QFD, 3QI5, 3QIR, 3QKD, 3QQK, 3QQN, 3QR2, 3QRI, 3QRJ, 3QRK, 3QTQ, 3QTR, 3QTS, 3QTU, 3QTW, 3QTX, 3QTZ, 3QU0, 3QX3, 3QXP, 3R0T, 3R43, 3R58, 3R6I, 3R7M, 3R7O, 3R85, 3R8G, 3R8H, 3R8I, 3R8U, 3R8V, 3R8Z, 3R94, 3R9D, 3R9H, 3R9N, 3R9O, 3RAH, 3RAK, 3RAL, 3RDH, 3RGF, 3RHK, 3RHX, 3RI1, 3RJ7, 3RJC, 3RK5, 3RK7, 3RK9, 3RKB, 3RKZ, 3RMF, 3RNI, 3RPR, 3RPV, 3RPY, 3RQD, 3RU0, 3RZ3, 3RZB, 3S00, 3S0O, 3S1H, 3S34, 3S35, 3S98, 3S9D, 3SA0, 3SBD, 3SDE, 3SE2, 3SI5, 3SL9, 3SMI, 3SMJ, 3SP7, 3SPF, 3SQQ, 3SWZ, 3SZ9, 3SZA, 3SZB, 3T1L, 3T1M, 3T2T, 3T6A, 3TB3, 3TE7, 3TEM, 3TF3, 3TH5, 3TH7, 3THE, 3THH, 3THJ, 3TJM, 3TNW, 3TSO, 3TUO, 3TZB, 3U15, 3U2Z, 3U5J, 3U5K, 3U5L, 3U6H, 3U6I, 3U6J, 3U9H, 3U9Y, 3UA9, 3UD1, 3UD2, 3UFY, 3UG8, 3UGC, 3UGR, 3ULI, 3UMW, 3UPA, 3UW4, 3UWP, 3V3B, 3V42, 3V5Q, 3V8S, 3VHA, 3VHC, 3VHD, 3VHE, 3VJN, 3VKX, 3VO3, 3VQU, 3VSO, 3W2O, 3W2P, 3W2Q, 3W2R, 3W2S, 3W51, 3WE4, 3WF5, 3WF6, 3WF7, 3WF8, 3WF9, 3WHA, 3WIX, 3WIY, 3WIZ, 3WUT, 3WZK, 3X01, 3X02, 3ZC6, 3ZCW, 3ZEP, 3ZFM, 3ZFY, 3ZGO, 3ZJB, 3ZK6, 3ZLN, 3ZLR, 3ZM0, 3ZM1, 3ZM2, 3ZM3, 3ZME, 3ZOS, 3ZVY, 3ZVZ, 3ZXE, 3ZXF, 3ZXZ, 3ZZE, 4A14, 4A1U, 4A1W, 4A5Y, 4A63, 4A69, 4A82, 4AG8, 4AGC, 4AGL, 4AGM, 4AGN, 4AGO, 4AGP, 4AGQ, 4AJP, 4AL0, 4AL1, 4AN3, 4ANB, 4AOI, 4AOW, 4AP7, 4APC, 4AQ3, 4AQC, 4AQI, 4AQJ, 4AS7, 4ASD, 4ASE, 4ASZ, 4AT3, 4AT4, 4AT5, 4AUV, 4AVM, 4AVP, 4AVU, 4AVW, 4AW9, 4AXA, 4AY1, 4AZA, 4B04, 4B2D, 4B36, 4B7P, 4B87, 4B9D, 4BCK, 4BCM, 4BCN, 4BCO, 4BCP, 4BCQ, 4BD8, 4BF2, 4BFP, 4BGH, 4BHN, 4BIB, 4BIE, 4BJ9, 4BJB, 4BJC, 4BJX, 4BKJ, 4BPM,

4BS4, 4BU3, 4BU5, 4BU6, 4BU7, 4BU8, 4BU9, 4BUA, 4BUE, 4BUI, 4BUS, 4BUT, 4BUU, 4BUV, 4BUW, 4BUX, 4BUY, 4C41, 4C4P, 4C52, 4C5D, 4CEG, 4CG8, 4CG9, 4CGA, 4CGB, 4CIM, 4CKR, 4CLI, 4CLJ, 4CMH, 4CMM, 4CMO, 4CMT, 4CMU, 4CNC, 4CNH, 4CNM, 4CQ0, 4CTB, 4CTC, 4D0N, 4D0W, 4D0X, 4D1S, 4D4M, 4DA5, 4DBS, 4DBW, 4DD8, 4DEA, 4DEE, 4DRA, 4DRH, 4DRI, 4DRJ, 4DRK, 4DRM, 4DRN, 4DRO, 4DRP, 4DSN, 4DSO, 4DST, 4DSU, 4E28, 4EDW, 4EEH, 4EEV, 4EEY, 4EFT, 4EFU, 4EGC, 4EHR, 4EJH, 4EJI, 4EJJ, 4EOI, 4EOJ, 4EOL, 4EOM, 4EON, 4EOO, 4EOP, 4EOQ, 4EOR, 4EQC, 4EQZ, 4ER3, 4ER6, 4ER7, 4EWI, 4F0E, 4F0I, 4F1L, 4FA3, 4FAL, 4FAM, 4FBX, 4FCJ, 4FCP, 4FCQ, 4FCR, 4FHH, 4FHI, 4FIA, 4FLP, 4FNW, 4FNX, 4FNY, 4FOB, 4FOC, 4FOD, 4FUL, 4FVL, 4FYQ, 4FYR, 4FYS, 4FYT, 4G1N, 4G31, 4GCJ, 4GDX, 4GG2, 4GJY, 4GJZ, 4GKS, 4GMX, 4GOS, 4GOV, 4GOY, 4GP3, 4GPZ, 4GS6, 4GS9, 4GT4, 4GT5, 4GV1, 4GWG, 4GWK, 4GZL, 4H1S, 4H1Y, 4H2B, 4H2F, 4H2G, 4H2I, 4H5S, 4H75, 4H7Y, 4H87, 4HBM, 4HC4, 4HG7, 4HJG, 4HKZ, 4HRF, 4HSG, 4HT1, 4HVC, 4HVS, 4HW3, 4HY4, 4HY5, 4I1F, 4I1H, 4I22, 4I24, 4I51, 4I7D, 4IA1, 4IAN, 4IBM, 4IBQ, 4IBS, 4IBT, 4IBU, 4IBV, 4IBW, 4IBY, 4IBZ, 4ICC, 4IEA, 4IEH, 4IFC, 4IFI, 4IGK, 4IHL, 4IIR, 4IJH, 4IJL, 4IJP, 4IJT, 4IKR, 4IKS, 4IKT, 4IKU, 4IOI, 4IPC, 4IPD, 4IPG, 4IPH, 4IRV, 4IS5, 4ISL, 4ISN, 4ISO, 4IU7, 4IUE, 4IUI, 4IV2, 4IV4, 4IVW, 4IVY, 4IW6, 4IW8, 4IWC, 4IWD, 4IWF, 4J0R, 4J0S, 4J1V, 4J1Z, 4J21, 4J22, 4J3L, 4J3M, 4J3Y, 4J44, 4J45, 4J46, 4J47, 4J48, 4J6G, 4J8M, 4J96, 4JA8, 4JBO, 4JBP, 4JBQ, 4JC1, 4JC3, 4JCK, 4JDD, 4JE4, 4JEG, 4JK5, 4JK6, 4JLK, 4JPG, 4JX7, 4JYG, 4JYI, 4JYW, 4JZ0, 4K0Y, 4K18, 4K1B, 4K1E, 4K4J, 4K6I, 4K7A, 4K80, 4K84, 4K85, 4K8Y, 4K9G, 4KAK, 4KBN, 4KBS, 4KCG, 4KCX, 4KFJ, 4KG8, 4KGA, 4KGQ, 4KJU, 4KJV, 4KJY, 4KM6, 4KM7, 4KMX, 4KMY, 4KMZ, 4KN0, 4KN1, 4KV1, 4KV4, 4KVP, 4KWP, 4KZL, 4KZO, 4KZQ, 4KZU, 4L03, 4L06, 4L09, 4L0B, 4L0I, 4L0P, 4L0S, 4L0T, 4L0V, 4L10, 4L2F, 4L2G, 4L2K, 4L31, 4L32, 4L33, 4L34, 4L4L, 4L4M, 4L5B, 4L8G, 4L8U, 4L8Z, 4L91, 4L94, 4L96, 4L98, 4L9K, 4L9S, 4L9W, 4LA0, 4LAV, 4LAW, 4LAX, 4LAY, 4LBJ, 4LBK, 4LBL, 4LBM, 4LBN, 4LBO, 4LGE, 4LGU, 4LOE, 4LOF, 4LP4, 4LPK, 4LQG, 4LRW, 4LUC, 4LUO, 4LUV, 4LUZ, 4LV6, 4LVT, 4LW1, 4LWC, 4LWE, 4LWF, 4LWG, 4LWH, 4LWI, 4LXD, 4LXZ, 4LY1, 4LYF, 4LYH, 4LYJ, 4M1O, 4M1S, 4M1T, 4M1W, 4M1Y, 4M21, 4M22, 4M2R, 4M2U, 4M2V, 4M2W, 4M8E, 4M8H, 4M9G, 4M9H, 4M9J, 4M9L, 4M9N, 4MA4, 4MAN, 4MCE, 4MCF, 4MDG, 4MDL, 4MDM, 4MDN, 4MDQ, 4MKC, 4MP2, 4MP7, 4MPC, 4MPE, 4MPN, 4MQ1, 4MSG, 4MSK, 4MT9, 4MTZ, 4MXC, 4MXO, 4MY6, 4MZF, 4MZG, 4MZH, 4MZI, 4N0S, 4N14, 4N16, 4N1T, 4N1U, 4N1Z, 4N20, 4N22, 4N24, 4N25, 4N26, 4N28, 4N2A, 4N2B, 4N2D, 4N2E, 4N2F, 4N2G, 4N2H, 4N2I, 4N2K, 4N2L, 4N2M, 4N2N, 4N3R, 4N4H, 4N4I, 4N4V, 4N5G, 4N6L, 4N6N, 4N6O, 4N8R, 4NB3, 4NDN, 4NFA, 4NGM, 4NGN, 4NGP, 4NGQ, 4NGR, 4NGS, 4NGT, 4NJ3, 4NKI, 4NMO, 4NMP, 4NMQ, 4NMR, 4NMS, 4NMT, 4NMV, 4NNX, 4NNY, 4NO2, 4NO3, 4NO5, 4NUC, 4NUD, 4NUE, 4NUS, 4O0A, 4O0C, 4O0D, 4O0E, 4O0F, 4O0G, 4O0H, 4O0R, 4O0X, 4O0Y, 4O0Z, 4O10, 4O12, 4O13, 4O14, 4O15, 4O16, 4O17, 4O18, 4O19, 4O1A, 4O1B, 4O1C, 4O1D, 4O1V, 4O28, 4O3M, 4O3N, 4O3O, 4O3P, 4O3Q, 4O3R, 4O3S, 4O70, 4O71, 4O72, 4O74, 4O75, 4O76, 4O77, 4O78, 4O7A, 4O7B, 4O7C, 4O7D, 4O7E, 4O7F, 4OA7, 4OAR, 4OAS, 4OC0, 4OC1, 4OC2, 4OC3, 4OC4, 4OC5, 4OEA, 4OEY, 4OEZ, 4OFR, 4OFU, 4OGI, 4OGJ, 4OH5, 4OHA, 4OJB, 4OJN, 4OK1, 4OKN, 4OKW, 4OKX, 4OMC, 4OO7, 4OOA, 4OQ6, 4OTD, 4OTH, 4OTI, 4OUL, 4OUM, 4OZ0, 4OZ1, 4P3C, 4P3D, 4P4C, 4P5E, 4P5Q, 4P5Z, 4P7U, 4PBZ, 4PCE, 4PCI, 4PGZ, 4PHJ, 4PHK, 4PHM, 4PIV, 4PNR, 4PO2, 4POH, 4POJ, 4PP3, 4PP5, 4PQW, 4PRY, 4PRZ, 4PS0, 4PS1, 4PS5, 4PTE, 4PTG, 4PVP, 4PVQ, 4PVR, 4PVS, 4PX9, 4PXM, 4PZR, 4PZS, 4PZY, 4PZZ, 4Q01, 4Q02, 4Q03, 4Q13, 4Q78, 4Q93, 4Q9V, 4QBT, 4QDQ, 4QDR, 4QDS, 4QEU, 4QEV, 4QEW, 4QG8, 4QG9, 4QGC, 4QIB, 4QL3, 4QML, 4QMM, 4QMN, 4QMO, 4QMP, 4QMQ, 4QMS, 4QMT, 4QMU, 4QMV, 4QMW, 4QMX, 4QMY, 4QMZ, 4QNA, 4QO9, 4QOC, 4QQK, 4QSA, 4QSB, 4QSI, 4QSJ, 4QSP, 4QSQ, 4QSR, 4QSS, 4QST, 4QSU, 4QSV, 4QSW, 4QSX, 4QTA, 4QTB, 4QTC, 4QTD, 4QTE, 4QUT, 4QUU, 4QVX, 4QXU, 4QXW, 4R0I, 4R3E, 4R59, 4R5A, 4R5B, 4R6E, 4R9A, 4R9B, 4R9C, 4R9D, 4RCI, 4RCJ, 4RDD, 4RE8, 4REE, 4REF, 4RFW, 4RG2, 4RJ3, 4RJF, 4RL7, 4RMD, 4RME, 4RMG, 4RMH, 4RMI, 4RMJ, 4RN0, 4RN1, 4RN2, 4ROC, 4ROE, 4RP6, 4RP7, 4RQI, 4RQR, 4RQX, 4RVK, 4RVL, 4RVM, 4RXA, 4S0O, 4TN7, 4TNB, 4TND, 4TNT, 4TOR, 4TOS, 4TPW, 4TQ9, 4TQA, 4TQB, 4TQC, 4TT6, 4TT7, 4TTE, 4TU4, 4TU6, 4TUH, 4TUJ, 4TUP, 4TUQ, 4TUR, 4TUS, 4TVJ, 4TW9, 4TWP, 4TYG, 4TYJ, 4TYL, 4TYO, 4TZ2, 4TZ8, 4U0I, 4U32, 4U6Q, 4U6S, 4U79, 4U7L, 4UAI, 4UAK, 4UAL, 4UAW, 4UAY, 4UAZ, 4UB1, 4UB3, 4UB4, 4UB5, 4UBB, 4UBC, 4UED, 4UHI, 4UMX, 4UMY, 4UND, 4UP0, 4UP5, 4UPG, 4USF, 4USL, 4UV7, 4UVL, 4UVN, 4UVO, 4UVP, 4UVS, 4UVT, 4UVU, 4UVV, 4UVW, 4UVX, 4UVY, 4UVZ, 4UWH, 4UWY, 4UX4, 4UXL, 4UYU, 4UYW, 4UZ1, 4UZ5, 4UZ6, 4UZ7, 4UZ9, 4UZA, 4UZL, 4UZQ, 4V0H, 4V26, 4W4Z, 4W50, 4W5I, 4W6E, 4W82, 4W9N, 4W9W, 4W9X, 4WA7, 4WA9, 4WB5, 4WB6, 4WB7, 4WB8, 4WBH, 4WDT, 4WDU, 4WDW, 4WDX, 4WEV, 4WFT, 4WHF, 4WHG, 4WIV, 4WJ5, 4WM0, 4WMA, 4WMB, 4WMI, 4WMK, 4WMR, 4WN2, 4WNO, 4WNP, 4WP7, 4WPN, 4WRB, 4WRS, 4WSQ, 4WT2, 4WUA, 4WUN, 4WUY, 4WV1, 4WVS, 4WVT, 4WVU, 4WZV, 4X0T, 4X0U, 4X2I, 4X3K, 4X3R, 4X3S, 4X4L, 4X5Y, 4X5Z, 4X60, 4X8Y, 4XCT, 4XCU, 4XDO, 4XDP, 4XEK, 4XEV, 4XH9, 4XI3, 4XMO, 4XO6, 4XO7, 4XT2, 4XV1, 4XV9, 4XVE, 4XYF, 4XZH, 4XZI, 4XZL, 4XZM, 4XZN, 4XZP, 4XZS, 4Y24, 4Y2E, 4Y2H, 4Y30, 4Y72, 4Y7R, 4Y85, 4Y8D, 4YAB, 4YAD, 4YAT, 4YAX, 4YBM, 4YBS, 4YBT, 4YC9, 4YH3, 4YH4, 4YJ4, 4YK0, 4YK5, 4YL0, 4YL1, 4YL3, 4YLZ, 4YM0, 4YM1, 4YM2, 4YM3, 4YMJ, 4YNE, 4YNM, 4YON, 4YPA, 4YPE, 4YPS, 4YQM, 4YQU, 4YQV, 4YTO, 4YVV, 4YVX, 4YWP, 4Z15, 4Z1Q, 4Z1S, 4Z1T, 4Z1U, 4Z3K, 4Z55, 4Z93, 4Z99, 4Z9A, 4Z9B, 4Z9O, 4Z9V, 4ZAO, 4ZBF, 4ZBK, 4ZC6, 4ZCG, 4ZFC, 4ZFI, 4ZG6, 4ZG7, 4ZGK, 4ZML, 4ZMO, 4ZMP, 4ZMQ, 4ZMV, 4ZMW, 4ZMY, 4ZMZ, 4ZN7, 4ZNH, 4ZNS, 4ZNT, 4ZNU, 4ZNV, 4ZNW, 4ZQK, 4ZRJ, 4ZRK, 4ZSA, 4ZSE, 4ZT1, 4ZTE, 4ZW1, 4ZWX, 4ZWY, 4ZWZ, 4ZX0, 4ZX1, 4ZXT, 4ZY5, 4ZY6, 4ZYF, 4ZYI, 4ZYT, 4ZYU, 4ZYV, 4ZYW, 4ZYX, 4ZYY, 4ZYZ, 4ZZ0, 4ZZX, 4ZZY, 4ZZZ, 5A19, 5A1F, 5A1G, 5A1I, 5A3N, 5A3P, 5A3T, 5A3W, 5A4K, 5A5N, 5A5O, 5A5P, 5A5Q, 5A5R, 5A5S, 5A6H, 5A7B, 5A7C, 5A7N, 5A7O, 5A7P, 5A7Q, 5A7S, 5A7W, 5A80, 5A81, 5A82, 5A83, 5A85, 5A9U, 5AA8, 5AA9, 5AAA, 5AAB, 5AAC, 5AAU, 5AAV, 5AB9, 5ABA, 5ACC, 5ADQ, 5ADR, 5ADS, 5ADT, 5AEA, 5AEH, 5AEP, 5AFV, 5AFW, 5AIZ, 5AJS, 5AKU, 5AKW, 5AL1, 5AL2, 5AL3, 5AL4, 5AL5, 5AM6, 5AM7, 5ANQ, 5ANS, 5ANT, 5ANU, 5ANV, 5ANW, 5AOI, 5AOJ, 5AOK, 5AOL, 5AOM, 5AP0, 5AP6, 5AP7, 5AQF, 5AQG, 5AQH, 5AQI, 5AQJ, 5AQK, 5AQL, 5AQM, 5AQN, 5AQO, 5AQP, 5AQR, 5AQS, 5AQT, 5AQU, 5AQV, 5AQW, 5AQX, 5AQY, 5ARF, 5ARG, 5AX9, 5AYF, 5B1Z, 5B6G, 5B7V, 5B8C, 5BK8, 5BOL, 5BOM, 5BON, 5BPC, 5BQG, 5BQH, 5BQI, 5BRO, 5BS0, 5BVK, 5BVN, 5BVO, 5BVW, 5BXJ, 5BXV, 5C26, 5C27, 5C37, 5C3T, 5C5P, 5C5Q, 5C5R, 5C5S, 5C67, 5C8N, 5CAP, 5CAS, 5CAU, 5CBL, 5CCW, 5CDB, 5CEI, 5CLS, 5COE, 5COI, 5CP5, 5CP9, 5CPE, 5CQD, 5CQH, 5CQI, 5CQK, 5CQT, 5CRM, 5CRZ, 5CS1, 5CS5, 5CS8, 5CS9, 5CSQ, 5CT1, 5CT2, 5CT3, 5CY9, 5CYJ, 5CZX, 5D0C, 5D24, 5D25, 5D26, 5D27, 5D3H, 5D3J, 5D3L, 5D3N, 5D3P, 5D3R, 5D3S, 5D3T, 5D3V, 5D3W, 5D3X, 5D3Y, 5D41, 5D5A, 5D6B, 5D6E, 5D6F, 5D7V, 5D8K, 5D8L, 5D9L, 5DA3, 5DAG, 5DAK, 5DAL, 5DBM, 5DCG, 5DDL, 5DET, 5DEY, 5DFP, 5DG2, 5DGH, 5DGI, 5DHJ, 5DI7, 5DIA, 5DID, 5DIE, 5DIG, 5DK3, 5DK9, 5DKB, 5DKG, 5DL4, 5DLR, 5DLS, 5DMC, 5DMF, 5DP0, 5DQ8, 5DQE, 5DRJ, 5DRM, 5DTV, 5DU5, 5DUE, 5DUG, 5DUH, 5DUU, 5DUV, 5DUW, 5DUX, 5DVS, 5DWE, 5DWG, 5DWI, 5DWJ, 5DWR, 5DX4, 5DXB, 5DXE, 5DXG, 5DXK, 5DXM, 5DXP, 5DXQ, 5DXR, 5DXT, 5DY4, 5DY5, 5DY8, 5DYB, 5DYD, 5DZ0,

5DZ1, 5DZ3, 5DZH, 5DZI, 5E0W, 5E0X, 5E14, 5E15, 5E19, 5E1B, 5E1C, 5E1D, 5E1M, 5E1O, 5E1S, 5E2A, 5E2B, 5E2Q, 5E33, 5E3A, 5E6H, 5E7N, 5E7R, 5E7V, 5E8M, 5E8S, 5E8T, 5E8U, 5E8V, 5E8W, 5E8X, 5E8Y, 5E8Z, 5E90, 5E91, 5E92, 5E97, 5E98, 5E9B, 5E9C, 5EA0, 5EA2, 5EAM, 5EAP, 5EAR, 5EBT, 5ECE, 5EFN, 5EH0, 5EHH, 5EHP, 5EHR, 5EHY, 5EI1, 5EI6, 5EI8, 5EIZ, 5EK3, 5EK9, 5EKI, 5EMV, 5EOB, 5EPB, 5EPK, 5EPL, 5EQ0, 5EU3, 5EU4, 5EU5, 5EU6, 5EVZ, 5EW8, 5EW9, 5EWS, 5EWZ, 5EX3, 5EX5, 5EXA, 5EXW, 5EY4, 5EYC, 5EYD, 5F0X, 5F1X, 5F2E, 5F2R, 5F36, 5F3A, 5F5Z, 5F60, 5F61, 5F62, 5F63, 5FA5, 5FC4, 5FCS, 5FCW, 5FDZ, 5FE0, 5FE1, 5FE2, 5FE3, 5FE4, 5FE5, 5FE6, 5FE7, 5FE8, 5FE9, 5FGK, 5FL4, 5FL5, 5FL6, 5FMI, 5FQP, 5FQR, 5FQS, 5FQT, 5FQV, 5FTO, 5FTQ, 5FUN, 5FUP, 5FUQ, 5FV8, 5FY4, 5FY5, 5FYB, 5FYS, 5FYT, 5FYU, 5FYV, 5FYY, 5FYZ, 5FZ0, 5FZ4, 5FZ6, 5FZ7, 5FZ8, 5FZ9, 5FZA, 5FZB, 5FZC, 5FZD, 5FZE, 5FZF, 5FZG, 5FZH, 5FZI, 5FZK, 5FZT, 5G01, 5G03, 5G0B, 5G0C, 5G15, 5G1X, 5G4M, 5G4N, 5G4O, 5GGQ, 5GGS, 5GGU, 5GGV, 5GHI, 5GHJ, 5GHM, 5GHN, 5GHO, 5GHP, 5GHQ, 5GNK, 5GP7, 5GS4, 5GTJ, 5GWN, 5GZC, 5GZD, 5GZE, 5GZF, 5GZG, 5H13, 5H14, 5H15, 5H17, 5H19, 5H1T, 5H1U, 5H1V, 5H2U, 5H43, 5H4J, 5H5Q, 5H5R, 5H5S, 5H65, 5H9P, 5H9Q, 5H9R, 5H9S, 5HBE, 5HCY, 5HES, 5HG5, 5HG7, 5HG8, 5HG9, 5HHH, 5HLW, 5HMH, 5HMI, 5HMK, 5HMP, 5HNI, 5HO6, 5HOA, 5HOR, 5HQ0, 5HVS, 5HVT, 5HYR, 5HZM, 5HZQ, 5I0H, 5I4H, 5I6V, 5I95, 5I96, 5IBM, 5IBS, 5ICP, 5IDN, 5IEV, 5IEX, 5IEY, 5IF4, 5IFS, 5IGK, 5IGL, 5IGM, 5IH8, 5IH9, 5IHA, 5IHC, 5IIS, 5IJR, 5ILS, 5ILU, 5ILV, 5IMX, 5IMY, 5INB, 5IRQ, 5ITA, 5IYY, 5IZ6, 5IZA, 5J1X, 5J28, 5J6A, 5J71, 5J7F, 5J7G, 5J7S, 5J89, 5J8I, 5J8O, 5J8R, 5JA4, 5JCU, 5JCW, 5JDS, 5JGA, 5JGI, 5JGQ, 5JH6, 5JHB, 5JHK, 5JI0, 5JJM, 5JK3, 5JMO, 5JMZ, 5JN3, 5JN7, 5JOG, 5JOH, 5JQ5, 5JQ8, 5JSJ, 5JSM, 5JSN, 5JXG, 5JXH, 5JXI, 5JXJ, 5JYM, 5JYO, 5JZJ, 5JZV, 5K0I, 5K31, 5K9V, 5K9W, 5KA0, 5KA1, 5KA2, 5KA3, 5KA4, 5KA7, 5KA8, 5KA9, 5KAA, 5KAB, 5KAC, 5KAD, 5KBR, 5KCC, 5KCD, 5KCE, 5KCF, 5KCT, 5KCU, 5KCW, 5KD9, 5KDH, 5KDI, 5KID, 5KJ2, 5KJH, 5KR9, 5KRA, 5KRC, 5KRF, 5KRH, 5KRI, 5KRK, 5KRL, 5KRM, 5KRO, 5KTU, 5KTW, 5KTX, 5KU3, 5KU9, 5KY6, 5KZ0, 5KZP, 5L11, 5L2S, 5L2T, 5L7A, 5L9B, 5L9R, 5L9V, 5LAP, 5LAS, 5LAT, 5LB6, 5LBB, 5LBC, 5LBE, 5LBF, 5LBW, 5LBY, 5LBZ, 5LC2, 5LE5, 5LEX, 5LEY, 5LEZ, 5LF0, 5LF1, 5LF3, 5LF4, 5LF6, 5LF7, 5LJ0, 5LJ1, 5LJ2, 5LK5, 5LN2, 5LNP, 5LOF, 5LPG, 5LPJ, 5LPK, 5LPL, 5LPM, 5LQB, 5LRQ, 5LW9, 5LWB, 5LX3, 5LX5, 5LX6, 5LXN, 5LXO, 5LY2, 5M2F, 5M39, 5M3A, 5M3V, 5M4C, 5M4E, 5M4F, 5M4H, 5M4I, 5M4M, 5M4P, 5M4U, 5M51, 5M53, 5M55, 5M56, 5M57, 5M5A, 5M5E, 5M9N, 5MAG, 5MAH, 5MAI, 5MAR, 5MC7, 5MES, 5MF6, 5MFP, 5MFZ, 5MGE, 5MGF, 5MGG, 5MGJ, 5MGK, 5MGN, 5MHQ, 5MIM, 5MKV, 5MKW, 5MKX, 5MKY, 5MKZ, 5ML5, 5MLE, 5MLI, 5MLJ, 5MLO, 5MLW, 5MPH, 5MPI, 5MPZ, 5MQE, 5MQG, 5MQK, 5MQV, 5MRB, 5MRD, 5MTJ, 5MTM, 5MWJ, 5MY6, 5MYG, 5N0M, 5N0Y, 5N1X, 5N1Z, 5N20, 5N21, 5N2D, 5N2F, 5N53, 5N5J, 5N5K, 5N84, 5N86, 5N87, 5N91, 5N93, 5N9C, 5N9K, 5N9L, 5N9N, 5N9P, 5N9R, 5N9S, 5N9T, 5NAJ, 5NBF, 5NBX, 5NC2, 5NCF, 5NCG, 5NCP, 5ND0, 5NDU, 5NEG, 5NFO, 5NGE, 5NGF, 5NGR, 5NGS, 5NGT, 5NJX, 5NJZ, 5NK0, 5NK1, 5NK2, 5NK3, 5NK4, 5NK5, 5NK6, 5NK7, 5NK8, 5NK9, 5NKA, 5NKB, 5NKC, 5NKD, 5NKE, 5NKF, 5NKG, 5NKH, 5NKI, 5NLK, 5NLV, 5NLY, 5NNC, 5NND, 5NNE, 5NNF, 5NNG, 5NOI, 5NP8, 5NQR, 5NR3, 5NRR, 5NRS, 5NRV, 5NRW, 5NSD, 5NSX, 5NT0, 5NT4, 5NU3, 5NU5, 5NUT, 5NV0, 5NV2, 5NVC, 5NVE, 5NVF, 5NVH, 5NVO, 5NWB, 5NWC, 5NWD, 5NWG, 5NX1, 5NX3, 5NXE, 5NZO, 5NZP, 5NZQ, 5O1A, 5O1B, 5O1C, 5O1D, 5O1E, 5O1F, 5O1G, 5O1H, 5O1I, 5O2D, 5O2T, 5O4Y, 5O7I, 5O9L, 5O9M, 5OA9, 5OAI, 5OC4, 5OC5, 5OC8, 5OCG, 5OCO, 5OCT, 5OFM, 5OFV, 5OFW, 5OGN, 5OGP, 5OI7, 5OKT, 5OM9, 5OMG, 5OMP, 5OMY, 5ONI, 5OOI, 5OQW, 5ORL, 5ORN, 5ORO, 5ORP, 5ORR, 5ORS, 5ORV, 5ORW, 5ORX, 5ORY, 5ORZ, 5OS0, 5OS1, 5OS2, 5OS3, 5OS4, 5OS5, 5OS6, 5OSD, 5OSE, 5OSF, 5OTE, 5OTF, 5OVB, 5OWH, 5OWL, 5OWM, 5OWS, 5OWT, 5OWW, 5OX5, 5OX6, 5QC7, 5QTZ, 5QU0, 5QXI, 5QXJ, 5QXK, 5QXL, 5QXM, 5QXN, 5QXO, 5QXP, 5QXQ, 5QXR, 5QXS, 5QXT, 5QXU, 5QXV, 5QXW, 5QXX, 5QXY, 5QXZ, 5QY0, 5R4E, 5R4F, 5R4V, 5R4W, 5R4X, 5R4Y, 5R4Z, 5SUN, 5SUY, 5SUZ, 5SVF, 5SVI, 5SVN, 5SVO, 5SVX, 5SVY, 5SXG, 5SXH, 5SXM, 5SYZ, 5SZ0, 5SZ1, 5SZ2, 5SZ3, 5SZ4, 5SZ5, 5SZ6, 5SZ7, 5T1Z, 5T36, 5T37, 5T3Q, 5T4U, 5T4V, 5T5J, 5T5L, 5T5P, 5T6P, 5T78, 5T92, 5TBM, 5TBX, 5TC4, 5TD5, 5TEG, 5TH6, 5TJ8, 5TKB, 5TKM, 5TL9, 5TLD, 5TLF, 5TLG, 5TLL, 5TLM, 5TLO, 5TLP, 5TLT, 5TLU, 5TLV, 5TLX, 5TLY, 5TM1, 5TM3, 5TM4, 5TM5, 5TM7, 5TM8, 5TML, 5TMM, 5TMO, 5TMQ, 5TMR, 5TMS, 5TMT, 5TMU, 5TMV, 5TMW, 5TMZ, 5TN1, 5TN4, 5TN5, 5TN6, 5TN7, 5TN9, 5TNB, 5TPT, 5TRF, 5TWL, 5TX4, 5TXS, 5TYI, 5TZ2, 5TZU, 5U06, 5U1Q, 5U28, 5U2B, 5U2D, 5U2E, 5U2J, 5U2M, 5U2N, 5U4X, 5U5P, 5U5R, 5U6C, 5U8G, 5U8H, 5U8I, 5UAB, 5UAD, 5UAT, 5UAU, 5UAV, 5UAW, 5UAX, 5UC4, 5UC6, 5UCJ, 5UE3, 5UE4, 5UEU, 5UEW, 5UEX, 5UEY, 5UEZ, 5UF0, 5UFP, 5UFW, 5UFX, 5UG8, 5UG9, 5UGA, 5UGC, 5UGH, 5UK9, 5ULN, 5UMC, 5UN7, 5UOO, 5UQW, 5UR1, 5US4, 5USH, 5USJ, 5UUK, 5UUL, 5UUM, 5UUP, 5UUT, 5UY8, 5UY9, 5UZ0, 5UZY, 5V02, 5V03, 5V37, 5V4Q, 5V5N, 5V60, 5V61, 5V62, 5V71, 5V7I, 5V7T, 5V9L, 5V9O, 5VBQ, 5VBR, 5VEB, 5VGI, 5VKC, 5VMP, 5VND, 5VR3, 5VTB, 5VX1, 5VXZ, 5VYK, 5W0G, 5W0H, 5W45, 5W49, 5W60, 5W61, 5W6O, 5W89, 5W8F, 5W8H, 5W8I, 5W8J, 5W8K, 5W8L, 5W9C, 5W9D, 5WBH, 5WDE, 5WDH, 5WEX, 5WG8, 5WGD, 5WGQ, 5WHH, 5WHI, 5WHR, 5WJ6, 5WMA, 5WMD, 5WMG, 5WMU, 5WNY, 5WNZ, 5WO0, 5WRV, 5WS7, 5WT9, 5X1V, 5X4M, 5X4N, 5X4O, 5X4P, 5X4Q, 5X4W, 5X4X, 5X4Y, 5X54, 5X5A, 5X5D, 5X5O, 5X66, 5X67, 5X7B, 5X93, 5X9O, 5X9P, 5XCO, 5XOC, 5XOD, 5XPI, 5XQZ, 5XU8, 5XVA, 5XVE, 5XVG, 5XXH, 5XXK, 5Y0C, 5Y0D, 5Y0Z, 5Y1Y, 5Y3N, 5Y5N, 5Y5T, 5Y5U, 5Y8C, 5Y8W, 5Y8Y, 5Y8Z, 5Y93, 5Y94, 5YDE, 5YDF, 5YE3, 5YE4, 5YGI, 5YJ8, 5YQL, 5YQM, 5YQN, 5YQO, 5YU9, 5YV8, 5YVB, 5YVC, 5Z0S, 5Z1R, 5Z1S, 5Z1T, 5Z30, 5ZCK, 5ZF4, 5ZF7, 5ZF8, 5ZF9, 5ZFA, 5ZFB, 5ZIB, 5ZIC, 5ZJD, 5ZQO, 5ZQP, 5ZQQ, 5ZQR, 5ZRF, 5ZTN, 6A1C, 6A5Q, 6A5S, 6A77, 6A78, 6A79, 6A7A, 6A7B, 6A84, 6AA3, 6AA4, 6AA5, 6AC9, 6AD9, 6AFR, 6AKO, 6AKP, 6AKW, 6ARJ, 6ARV, 6AU6, 6AVF, 6AVJ, 6AVZ, 6AW0, 6AW1, 6B4L, 6B4U, 6B59, 6B5A, 6B94, 6BA2, 6BA4, 6BA5, 6BAN, 6BBS, 6BC9, 6BCA, 6BCC, 6BEV, 6BG6, 6BIT, 6BLA, 6BMR, 6BMU, 6BMV, 6BMW, 6BMX, 6BMY, 6BOF, 6BR1, 6BSB, 6BSC, 6BUU, 6BXY, 6BY8, 6BYY, 6C2R, 6C2T, 6C42, 6C48, 6C4S, 6C6N, 6C7Q, 6C7R, 6CCR, 6CCT, 6CCU, 6CD8, 6CD9, 6CDC, 6CDG, 6CDR, 6CEH, 6CG1, 6CG2, 6CHW, 6CHZ, 6CMP, 6CMR, 6CPS, 6CQD, 6CQE, 6CQF, 6CRH, 6CT2, 6CT9, 6CU6, 6CUO, 6CUP, 6CUR, 6CWU, 6CWV, 6CWY, 6CYG, 6CYH, 6CZ3, 6CZ4, 6CZK, 6CZS, 6CZU, 6CZV, 6D12, 6D3F, 6D4D, 6D4F, 6D4P, 6D65, 6D68, 6D9X, 6DAI, 6DAK, 6DAR, 6DAS, 6DBP, 6DD3, 6DDH, 6DDI, 6DDJ, 6DDL, 6DDM, 6DDO, 6DDQ, 6DDR, 6DDV, 6DDY, 6DDZ, 6DE0, 6DE1, 6DE2, 6DE4, 6DFD, 6DFN, 6DHU, 6DIE, 6DIH, 6DIM, 6DJC, 6DJD, 6DJE, 6DJF, 6DJG, 6DJH, 6DJI, 6DJJ, 6DK5, 6DL2, 6DL7, 6DMG, 6DMI, 6DMJ, 6DMK, 6DML, 6DNB, 6DND, 6DUK, 6DUM, 6DUN, 6DXG, 6DXL, 6DY7, 6DYZ, 6DZ0, 6DZ2, 6DZ3, 6DZH, 6E1Y, 6E1Z, 6E22, 6E23, 6E3I, 6E3J, 6E40, 6E41, 6E42, 6E43, 6E44, 6E45, 6E46, 6E4F, 6E5G, 6E6C, 6E6E, 6E6G, 6E6H, 6E6J, 6E6P, 6E8C, 6E8P, 6E8X, 6E91, 6E92, 6EB6, 6EBE, 6ECZ, 6EDA, 6EEA, 6EEH, 6EEO, 6EG9, 6EGD, 6EGE, 6EH4, 6EH5, 6EH6, 6EH7, 6EH9, 6EI8, 6EIN, 6EIU, 6EIY, 6EJ0, 6EJ1, 6EK6, 6EOP, 6EPJ, 6EPR, 6EPS, 6EPT, 6EPU, 6EPV, 6EPW, 6EPX, 6EQV, 6EQW, 6EQX, 6ET4, 6ETE, 6ETG, 6ETS, 6ETT, 6ETU, 6ETV, 6ETW, 6EXW, 6EZA, 6EZB, 6EZC, 6F00, 6F0A, 6F0F, 6F0G, 6F0H, 6F1X, 6F20, 6F22, 6F23, 6F2U, 6F3B, 6F3D, 6F3G, 6F3I, 6F4M, 6F4O, 6F4P, 6F4Q, 6F4R, 6F4S, 6F4T, 6F5Q, 6F5R, 6F5S, 6F5T, 6F5X, 6F62, 6F63, 6F64, 6F78, 6F7B, 6F7H, 6FA1, 6FA3, 6FA4, 6FAF, 6FAG, 6FAU, 6FAV, 6FAW, 6FBK, 6FBO, 6FBP, 6FBW,

6FBY, 6FC8, 6FCD, 6FCF, 6FCK, 6FE0, 6FE1, 6FE2, 6FF9, 6FFJ, 6FG1, 6FG6, 6FGG, 6FGH, 6FGL, 6FGT, 6FGU, 6FH6, 6FH7, 6FI4, 6FI5, 6FIS, 6FIW, 6FJI, 6FJJ, 6FNF, 6FNG, 6FNH, 6FNI, 6FNJ, 6FNK, 6FNL, 6FNM, 6FO8, 6FQ1, 6FQR, 6FR3, 6FR4, 6FR5, 6FR7, 6FR8, 6FR9, 6FRA, 6FRB, 6FRC, 6FS0, 6FS1, 6FT7, 6FT8, 6FT9, 6FUM, 6FUN, 6FUO, 6FUP, 6FUQ, 6FUR, 6FVF, 6FVG, 6FWB, 6FXH, 6FZF, 6FZG, 6FZJ, 6FZP, 6FZQ, 6FZR, 6G0O, 6G0P, 6G0Q, 6G0R, 6G0S, 6G24, 6G25, 6G27, 6G29, 6G2B, 6G2C, 6G2E, 6G2F, 6G2O, 6G2V, 6G2X, 6G2Y, 6G2Z, 6G30, 6G5W, 6G5X, 6G6J, 6G6K, 6G6L, 6G6R, 6G98, 6G9U, 6GCY, 6GDY, 6GFA, 6GG1, 6GGA, 6GGB, 6GGC, 6GGD, 6GGE, 6GGF, 6GGN, 6GI6, 6GIN, 6GIP, 6GJW, 6GL8, 6GQE, 6GT3, 6GU2, 6GU6, 6GUC, 6GUE, 6GUH, 6GUK, 6GVA, 6GVX, 6GX6, 6GXB, 6GXE, 6GXK, 6GY5, 6H0W, 6H0X, 6H0Y, 6H0Z, 6H10, 6H11, 6H1D, 6H1E, 6H3K, 6H3Q, 6H46, 6H47, 6H4K, 6H4Z, 6H50, 6H51, 6H52, 6HAX, 6HAY, 6HAZ, 6HBN, 6HBY, 6HCZ, 6HDN, 6HDQ, 6HHP, 6HI3, 6HIA, 6HIE, 6HJ2, 6HKB, 6HKF, 6HKQ, 6HMB, 6HMC, 6HMD, 6HME, 6HMT, 6HMU, 6HN2, 6HN3, 6HNW, 6HNY, 6HOP, 6HOQ, 6HOR, 6HOT, 6HOU, 6HOV, 6HP9, 6HPH, 6HPQ, 6HPT, 6HQU, 6HR2, 6HVH, 6HVI, 6HVJ, 6HXK, 6HXL, 6HXM, 6HYG, 6HYJ, 6HYY, 6I07, 6I0J, 6I0L, 6I0Z, 6I10, 6I11, 6I12, 6I13, 6I14, 6I15, 6I16, 6I17, 6I18, 6I1S, 6I3S, 6I41, 6I5N, 6I5P, 6I68, 6I6Z, 6I7A, 6I7Q, 6I7R, 6I8Y, 6I9L, 6I9M, 6I9N, 6IAR, 6IB2, 6IBX, 6IBZ, 6IE3, 6IIE, 6IJX, 6ILI, 6IMZ, 6INL, 6IO0, 6IQ6, 6ITJ, 6IUO, 6IUP, 6IVZ, 6IWD, 6IWV, 6IZQ, 6J1L, 6J1X, 6J3B, 6J3C, 6J4O, 6J4P, 6J4U, 6J4V, 6J6Y, 6J8Y, 6JD0, 6JD8, 6JIB, 6JMD, 6JME, 6JPV, 6JTN, 6JTP, 6JX5, 6JZC, 6JZD, 6K0J, 6K0T, 6K0Y, 6K39, 6K95, 6KG2, 6KLA, 6KN5, 6KOF, 6KPS, 6KRO, 6KSQ, 6KX1, 6KX2, 6KX3, 6KZC, 6KZD, 6L0X, 6L10, 6L1C, 6L1F, 6L1I, 6L1P, 6L3R, 6L4B, 6L9K, 6L9L, 6LIH, 6LIL, 6LIO, 6LP6, 6LP7, 6LP8, 6LPF, 6LR9, 6LS5, 6LUB, 6LUD, 6LVK, 6LXY, 6M64, 6MAJ, 6MAK, 6MAU, 6MAV, 6MD7, 6MDB, 6MGN, 6MH1, 6MH7, 6MHC, 6MHD, 6MIL, 6MIN, 6MIO, 6MIQ, 6MJ5, 6MK3, 6MKK, 6MKM, 6MKO, 6MLC, 6MNX, 6MOB, 6MVL, 6MWE, 6MX8, 6MXO, 6MYZ, 6MZ0, 6MZQ, 6MZW, 6N0D, 6N0N, 6N0O, 6N0R, 6N17, 6N19, 6N2J, 6N2K, 6N3G, 6N3O, 6N4T, 6N8X, 6N8Y, 6NE5, 6NE7, 6NEC, 6NFK, 6NFL, 6NFY, 6NG0, 6NG1, 6NG2, 6NG4, 6NG5, 6NG6, 6NG7, 6NG8, 6NGA, 6NGB, 6NGC, 6NGD, 6NGE, 6NGF, 6NGH, 6NGI, 6NH1, 6NH2, 6NH3, 6NH4, 6NH5, 6NH6, 6NH7, 6NH8, 6NHB, 6NHC, 6NHF, 6NJA, 6NJZ, 6NK0, 6NK1, 6NK2, 6NKP, 6NKR, 6NKS, 6NKU, 6NKV, 6NKW, 6NKX, 6NKY, 6NKZ, 6NL0, 6NLV, 6NM0, 6NM7, 6NMR, 6NMS, 6NMT, 6NMW, 6NNG, 6NNV, 6NOJ, 6NP9, 6NPZ, 6NRF, 6NRG, 6NRH, 6NRI, 6NRJ, 6NT2, 6NTP, 6NTU, 6NU1, 6NU5, 6NUB, 6NVB, 6NWS, 6NWT, 6O0K, 6O0L, 6O0M, 6O0O, 6O0P, 6O1E, 6O39, 6O3A, 6O4U, 6O4Y, 6O4Z, 6O51, 6O53, 6O5M, 6O6F, 6O6G, 6O89, 6O9B, 6O9C, 6O9X, 6O9Y, 6OA0, 6OA1, 6OA3, 6OAK, 6OAL, 6OB3, 6OCX, 6OD0, 6OIA, 6OIE, 6OIL, 6OIM, 6OKD, 6OLX, 6ONY, 6OPD, 6OQB, 6OQC, 6OQD, 6OQN, 6OSH, 6OSN, 6OSP, 6OSV, 6OTN, 6OWH, 6OWI, 6P05, 6P1D, 6P3D, 6P3P, 6P4V, 6P5S, 6P6D, 6P8W, 6P8X, 6P8Y, 6P8Z, 6PDA, 6PDB, 6PDC, 6PDD, 6PDE, 6PDF, 6PDG, 6PEG, 6PET, 6PF1, 6PG3, 6PG4, 6PG5, 6PG6, 6PG7, 6PG8, 6PG9, 6PGA, 6PGB, 6PGC, 6PGD, 6PGE, 6PGF, 6PGR, 6PGU, 6PGX, 6PJV, 6PKC, 6PLF, 6PPA, 6PQ3, 6PTB, 6PTE, 6PU7, 6PV9, 6PVE, 6PYY, 6PYZ, 6Q0D, 6Q13, 6Q38, 6Q4Q, 6Q6J, 6Q8K, 6QAS, 6QAU, 6QAV, 6QB3, 6QB4, 6QB6, 6QB9, 6QBC, 6QBY, 6QCD, 6QCE, 6QCH, 6QCI, 6QCJ, 6QCN, 6QED, 6QEF, 6QEG, 6QEH, 6QEI, 6QEJ, 6QEY, 6QF9, 6QFC, 6QFQ, 6QGG, 6QGH, 6QGJ, 6QGK, 6QGV, 6QPL, 6QTJ, 6QUU, 6QUV, 6QUW, 6QUX, 6QVG, 6QVL, 6QXJ, 6QYK, 6QYL, 6QYO, 6QYP, 6QYQ, 6QZ5, 6QZ6, 6QZ7, 6QZ8, 6QZB, 6QZH, 6R2L, 6R3E, 6R3S, 6R9H, 6RB1, 6RBC, 6RBI, 6RCB, 6RCF, 6RCM, 6RFE, 6RFF, 6RFI, 6RLC, 6RLW, 6RNU, 6ROL, 6ROY, 6RQ4, 6RTI, 6RWJ, 6RZ6, 6RZ7, 6S1X, 6S4B, 6S55, 6S56, 6S57, 6S6K, 6S70, 6S71, 6S74, 6S77, 6S79, 6S7A, 6S7C, 6S7F, 6S7H, 6S8L, 6S8V, 6SA2, 6SA3, 6SAH, 6SAJ, 6SB8, 6SBO, 6SCM, 6SD9, 6SDC, 6SDD, 6SDE, 6SEQ, 6SER, 6SF3, 6SFI, 6SFJ, 6SFK, 6SFR, 6SG4, 6SGD, 6SGI, 6SGK, 6SHH, 6SHI, 6SHZ, 6SI0, 6SI1, 6SI2, 6SI3, 6SI4, 6SJU, 6SK9, 6SLG, 6SM8, 6SMB, 6SPW, 6SPX, 6SQC, 6ST3, 6SWN, 6SWQ, 6SZM, 6T1I, 6T1J, 6T1L, 6T1M, 6T1N, 6T1O, 6T41, 6T6R, 6T7U, 6T9Z, 6TD4, 6TE6, 6TEL, 6TEN, 6TFU, 6TFV, 6TFW, 6TFY, 6TFZ, 6TG0, 6TG1, 6THW, 6THX, 6THZ, 6TI8, 6TKP, 6TMO, 6TMP, 6TMQ, 6TN0, 6TN2, 6TN4, 6TN5, 6TNC, 6TOF, 6TOG, 6TOH, 6TOI, 6TOJ, 6TOK, 6TOL, 6TOM, 6TON, 6TOO, 6TRN, 6TVE, 6TVG, 6TWA, 6TX0, 6TX2, 6TXF, 6U0A, 6U15, 6U16, 6U17, 6U1U, 6U4Q, 6U4T, 6U65, 6U67, 6U6V, 6U73, 6U8Z, 6UBW, 6UCS, 6UD2, 6UDI, 6UDT, 6UDU, 6UDV, 6UDX, 6UDY, 6UEL, 6UFX, 6UGN, 6UGO, 6UGP, 6UGQ, 6UGR, 6UGZ, 6UH0, 6UJO, 6UKM, 6UKU, 6UKV, 6UKW, 6UKX, 6UKY, 6UKZ, 6UL0, 6ULI, 6ULK, 6ULM, 6ULN, 6UMT, 6UMU, 6UMV, 6UNT, 6URE, 6URJ, 6URK, 6URL, 6URN, 6URQ, 6URR, 6URZ, 6US0, 6US1, 6US7, 6USB, 6USD, 6USI, 6USX, 6USZ, 6UT0, 6UYA, 6UZF, 6UZT, 6UZU, 6V0Q, 6V0S, 6V0U, 6V0X, 6V14, 6V16, 6V17, 6V1B, 6V1D, 6V1E, 6V1F, 6V1H, 6V1K, 6V1L, 6V1U, 6V3W, 6V52, 6V5N, 6V5P, 6V66, 6V6K, 6V6O, 6V74, 6V7C, 6V7D, 6V7E, 6V7F, 6V7Y, 6V87, 6V89, 6V8A, 6V8K, 6V8N, 6V8T, 6V90, 6V9C, 6VBH, 6VC9, 6VEI, 6VFZ, 6VGH, 6VGO, 6VHG, 6VHI, 6VHN, 6VIG, 6VKG, 6VKK, 6VMU, 6VND, 6VO4, 6VO7, 6VPF, 6VPK, 6VQN, 6VR1, 6VR5, 6VRN, 6VTC, 6VTH, 6VUA, 6W0Q, 6W2P, 6W3Q, 6W3U, 6W43, 6W4R, 6W4Z, 6W7F, 6W7G, 6W7J, 6W7K, 6W7L, 6WBE, 6WE3, 6WE4, 6WGH, 6WGN, 6WH1, 6WH2, 6WHB, 6WKA, 6WP3, 6WP4, 6WP5, 6WP6, 6WU8, 6WUW, 6WW3, 6WW4, 6WW8, 6X2M, 6X2P, 6X2R, 6X2S, 6X2U, 6X2X, 6X2Y, 6X3N, 6X3O, 6X3P, 6X7B, 6XDL, 6XLW, 6XLX, 6XOG, 6XOH, 6XOI, 6XOZ, 6XP0, 6XP1, 6XP2, 6XP3, 6XPB, 6XPC, 6XU1, 6XUH, 6XUI, 6XUL, 6XUN, 6XVT, 6XVW, 6XXH, 6XXI, 6XXJ, 6XXK, 6XXL, 6XXM, 6XXR, 6Y09, 6Y1J, 6Y4S, 6Y74, 6Y7Q, 6Y8K, 6YAU, 6YB4, 6YB8, 6YB9, 6YBG, 6YBK, 6YBL, 6YG9, 6YHR, 6YKD, 6YLI, 6YQN, 6YQO, 6YQP, 6YVW, 6YVX, 6YVZ, 6YW0, 6YW1, 6YW2, 6YW3, 6YW4, 6YZJ, 6YZL, 6YZM, 6YZO, 6YZP, 6YZQ, 6YZR, 6YZS, 6YZT, 6YZU, 6YZV, 6YZW, 6YZX, 6Z04, 6Z4D, 6Z83, 6Z9B, 6ZAY, 6ZDC, 6ZDK, 6ZDL, 6ZFA, 6ZFN, 6ZFQ, 6ZHC, 6ZJ1, 6ZJ5, 6ZK6, 6ZRV, 7A04, 7A06, 7A1B, 7A1Z, 7A22, 7A2A, 7A2H, 7A49, 7A4B, 7A62, 7A77, 7A78, 7A79, 7A9Y, 7A9Z, 7AA0, 7AA1, 7ACK, 7AD0, 7AD7, 7AH1, 7AH4, 7AHZ, 7AI0, 7AI1, 7AIA, 7AKI, 7AQF, 7AQG, 7ASU, 7ASV, 7AVQ, 7AVX, 7AVY, 7AVZ, 7AW0, 7AW1, 7AW2, 7AW3, 7AW4, 7B3M, 7B7R, 7B94, 7B9L, 7BCM, 7BKG, 7BOS, 7BOT, 7BSJ, 7C04, 7C7F, 7C7G, 7C7H, 7CNA, 7CTP, 7CYZ, 7D3D, 7DHY, 7DHZ, 7JHD, 7JJM, 7JK7, 7JOO, 7JU5, 7JU6, 7JV8, 7JVM, 7JVN, 7JWS, 7JWT, 7JWU, 7JWV, 7JWW, 7JX9, 7K6I, 7K6J, 7K6K, 7K6L, 7K6M, 7K6T, 7K6U, 7K6X, 7K6Z, 7KBS, 7KCA, 7KCD, 7KIA, 7KIE, 7KK2, 7KK3, 7KK4, 7KK5, 7KK6, 7KKM, 7KKN, 7KKO, 7KKP, 7KKQ, 7KWM, 7LSZ, 7LT0, 7NBJ, 7NBQ

Table S2. Static and flexible docking analysis of chemical structures with molecular similarity to PSK and PSP.

Ligand	Structure	Molecular properties	Docking	Affinity	EL	HB amino acids	Covalent amino acids (pi-alkyl, Pi-Pi)	VW amino acids
Adenosine monophosphate	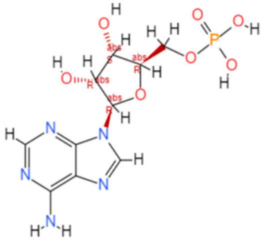	miLogP:-1.52<br>TPSA:186.08<br>Natoms:23<br>MW:347.22g/mol<br>nON:12<br>nOHNH:6<br>nviolations:2<br>nrotb:4<br>volume:263.88	Estático	-8.2	-0.35	ASP36, ASN117, HIS118, HIS243, GLY392, ARG395, ASP506	PHE417, PHE500	ASN245, ARG354, ASN390, GLY392, GLY393, THR446
			Flexible	-7.0	-0.3	HIS118, HIS243		LEU184, SER244, ASN245, GLY392, GLY393, ARG395, THR446, GLY447, PHE500, ASP506
AB680	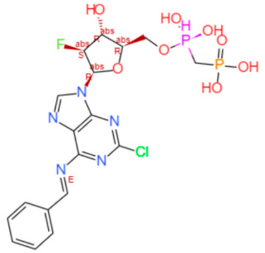	miLogP:0.27<br>TPSA:189.16<br>Natoms:35<br>MW:578.81 g/mol<br>nON:13<br>nOHNH:5<br>nviolations:2<br>nrotb:9<br>volume:440.10	Static	-7.3	-0.2	GLU180, HIS440, ARG 441, HIS437, ASP506, GLY507	MET510, HIS437, HIS440	LYS433, GLN509, ASP513, GLU514
			Flexible	-6.7	-0.19	LEU184, ASN186	HIS118, ASN 122, GLY123, SER185, ASN390, GLY393, PHE417, GLY418	ASP121, PHE500
CVP	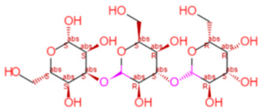	miLogP:-5.25<br>TPSA:268.68<br>Natoms:34<br>MW:483.27g/mol<br>nON:16<br>nOHNH:11<br>nviolations:3<br>nrotb:7<br>volume:352.07	Static	-7.2	-0.21	GLU180, ARG395, ARG441, GLN444, THR446, GLY447, ASP506		HIS118. GLU224, HIS243, SER244, ASN245, ASN390, GLY392, SER445, PHE500
			Flexible	-6.8	-0.2	TYR 345, ASP347, GLU400, ARG401, VAL542		LYS256, VAL344, SER352, ASN359, ASP399, PRO540, ALA541

Table S2. Static and flexible docking analysis of chemical structures with molecular similarity to PSK and PSP.

Ligand	Structure	Molecular properties	Docking	Affinity	EL	HB amino acids	Covalent amino acids (pi-alkyl, Pi-Pi)	VW amino acids
3-beta-Glucosylcellotriose	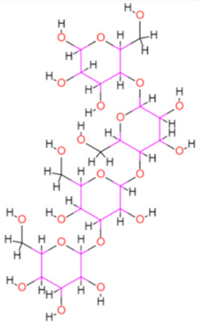	miLogP:-5.69<br>TPSA:347.83<br>Natoms:45<br>MW:666.58 g/mol<br>nON:21<br>nOHNH:14<br>nviolations:3<br>nrotb:10<br>volume:548.17	Static	-6.8	-0.15	GLU180, ARG441, HIS440, GLN444		GLU224, HIS243, THR446, GLY447, GLY507
			Flexible	-6.5	-0.14	ASP366, TYR484, GLU543		ASN362, ASN370, GLY454, HIS456, LEU475, TYR531, LYS534, MET535, PRO540, ARG545
Glycogen	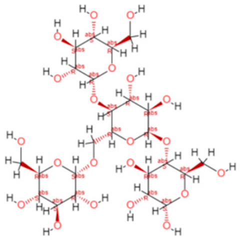	miLogP:-5.65<br>TPSA:347.83<br>Natoms:45<br>MW:666.58<br>nON:21<br>nOHNH:14<br>nviolations:3<br>nrotb:10<br>volume:548.17	Static	-6.8	-0.15	GLU180, ARG441, GLN444		LEU184, GLU224, SER244, HIS437, THR446, ASP506, GLY507
			Flexible	-6.9	-0.15	GLU180		LEU 184, MET225, HIS243, HIS437, HIS440, ARG441, THR446, GLY447, GLU448, ASP506, GLY507, GLN509
Laminarin 2	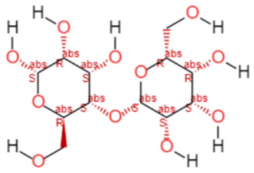	miLogP:-4.45<br>TPSA:189.53<br>Natoms:23<br>MW:342.30g/mol<br>nON:11<br>nOHNH:8<br>nviolations:2	Static	-6.7	-0.29	HIS243, ASN390, GLY393		ASP36, ASP85, ASN117, HIS118, LEU184, SER244, ASN245, ARG354, GLY392, GLY393, ARG395, PHE417, GLY447, ASP506

Table S2. Static and flexible docking analysis of chemical structures with molecular similarity to PSK and PSP.

Ligand	Structure	Molecular properties	Docking	Affinity	EL	HB amino acids	Covalent amino acids (pi-alkyl, Pi-Pi)	VW amino acids
		nrotb:4<br>volume:283.93	Flexible	-6.8	-0.29	GLU180		PHE183, LEU184, GLU224, HIS437, HIS440, ARG441, ASP506, GLY507
Laminarin 3	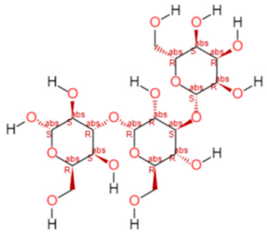	miLogP:-5.25<br>TPSA:268.68<br>Natoms:34<br>MW:504.44g/mol	Static	-5.8	-0.17	GLU224, ARG441, GLN444, THR446		HIS243, SER244, ARG354, SER445, GLY447
		nON:16<br>nOHNH:11<br>nviolations:3<br>nrotb:7<br>volume:416.05	Flexible	-7.2	-0.21	ARG441, GLY509		PHE 183, LEU184, GLU224, HIS437, HIS440, ARG 441, ASP506, GLY507
Laminarin 4	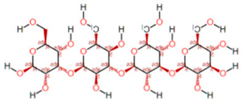	miLogP:-5.92<br>TPSA:347.83<br>Natoms:45<br>MW:638.36 g/mol	Static	-6.4	-0.14	GLU180, GLN509		PHE183, LEU184, HIS243, HIS437, HIS440, ARG441, THR446, ASP506, GLY507
		nON:21<br>nOHNH:14<br>nviolations:3<br>nrotb:10<br>volume:462.87	Flexible	-4.8	-0.11	GLU180, ARG441		GLU224, HIS437, GLN444, ASP506, GLY507, GLN509, MET510
Laminarin 6	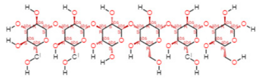	miLogP:-6.42<br>TPSA:506.13<br>Natoms:67<br>MW:948.52 g/mol<br>nON:31	Static	-7.2	-0.11	ARG354, LYS433, HIS437, HIS440 GLN444, GLY507, ASP513		GLU180, GLU224, SER244, ARG441, SER445, THR446, GLY447, ASP506 GLN509, MET510 GLU514
		nOHNH:20<br>nviolations:3<br>nrotb:16	Flexible	-6.1	-0.09	GLN444<br>ARG468<br>GLY507		GLU180, GLU224, LYS227, HIS437,

Table S2. Static and flexible docking analysis of chemical structures with molecular similarity to PSK and PSP.

Ligand	Structure	Molecular properties	Docking	Affinity	EL	HB amino acids	Covalent amino acids (pi-alkyl, Pi-Pi)	VW amino acids
		volume:684.46						HIS440, ARG441, TYR442
Laminarin 7	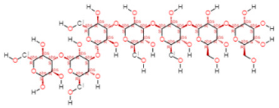	miLogP:-6.60<br>TPSA:585.29<br>Natoms:78<br>MW:1103.61<br>nON:36<br>nOHNH:23<br>nviolations:3<br>nrotb:19<br>volume:795.25	Static	-7.9	-0.10	GLU180, LYS433, HIS437, ARG441		HIS118, LEU184, GLU224, SER244, ASN245, GLY352, ARG354, ASN390, ARG395, HIS440, GLN444, SER445, THR446, GLY447, PHE500, ASP506, GLY507, GLN509, MET510, ASP513
			Flexible	-5.7	-0.09	GLU180, HIS440, ARG441, GLN444, GLY507		GLU224, HIS437, ASP506, GLN509, MET510
Sizofiran	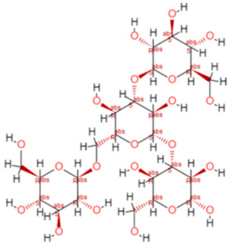	miLogP:-5.65<br>TPSA:347.83<br>Natoms:45<br>MW:666.58g/mol<br>nON:21<br>nOHNH:14<br>nviolations:3<br>nrotb:10<br>volume:548.17	Static	-6.7	-0.14	ARG441, GLN444, ASP506		GLU118, LEU184, GLU224, SER244, HIS437, HIS440, THR446, GLU448, HIS437
			Flexible	-6.5	-0.13	HIS440, ARG441, GLY502, GLN509		PHE183, HIS437
PSK		miLogP:-6.13<br>TPSA:485.91<br>Natoms:66<br>MW:931.52 g/mol	Static	-6.3	-<br>0.095	GLU180, ARG441, GLY507, MET510		PHE183, LEU184, GLU224, HIS243, SER244, HIS37, HIS440, THR446, GLU500

Table S2. Static and flexible docking analysis of chemical structures with molecular similarity to PSK and PSP.

Ligand	Structure	Molecular properties	Docking	Affinity	EL	HB amino acids	Covalent amino acids (pi-alkyl, Pi-Pi)	VW amino acids
	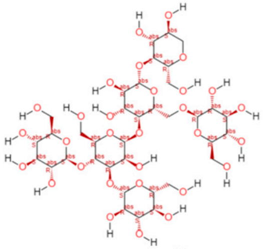	nON:30<br>nOHNH:19<br>nviolations:3<br>nrotb:16<br>volume:673.80	Flexible	-6.8	-0.1	GLU180, ARG441, GLN444, GLN509		PHE183, GLU224, HIS437, HIS440, GLY507
Malt triose	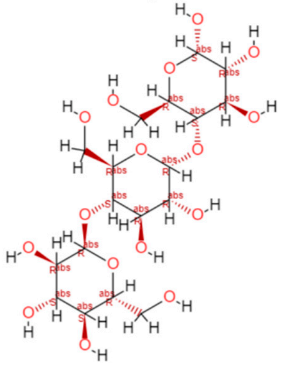	miLogP:-5.25<br>TPSA:268.68<br>Natoms:34<br>MW:504.44g/mol<br>nON:16<br>nOHNH:11<br>nviolations:3<br>nrotb:7<br>volume:416.05	Static	-6	-0.18	GLU180, ARG441, GLN444		PHE183, LEU184, GLU224, HIS243, SER244, HIS37, HIS440, THR446, GLU500
			Flexible	-6.5	-0.19	GLU180, ARG441, GLN444		GLU224, HIS243, HIS437, ASP506, GLY507
Beta glucan		miLogP:-5.25<br>TPSA:268.68<br>Natoms:34<br>MW:504.44 g/mol<br>nON:16<br>nOHNH:11<br>nviolations:3	Static	-5.6	-0.17	ARG441, GLN444		GLU180, GLU224, LYS227, HIS243, THR446

Table S2. Static and flexible docking analysis of chemical structures with molecular similarity to PSK and PSP.

Ligand	Structure	Molecular properties	Docking	Affinity	EL	HB amino acids	Covalent amino acids (pi-alkyl, Pi-Pi)	VW amino acids
	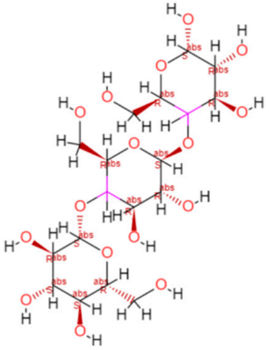	nrotb:7<br>volume:416.05	Flexible	-7.5	-0.23	GLY507		GLU180, HIS243, HIS440, THR446, GLY447, GLN509
CVG	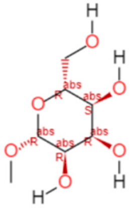	miLogP:-2.03<br>TPSA:99.38<br>Natoms:13<br>MW:194.18g/mol<br>nON:6<br>nOHNH:4<br>nviolations:0<br>nrotb:2<br>volume:169.34	Static	-5.5	-0.42	ARG354, ARG395, GLY447		HIS38, ASP85, ASN117, HIS118, HIS243, ASN245, GLY392
			Flexible	-6.4	-0.49	ARG354, GLY392, ASP506		HIS118, LEU184, HIS243, ASN390, GLY393, ARG395, SER445, THR446, GLY447, GLU448, PHE500
